# Extensive intra-phylotype diversity in lactobacilli and bifidobacteria from the honeybee gut

**DOI:** 10.1186/s12864-015-1476-6

**Published:** 2015-04-11

**Authors:** Kirsten M Ellegaard, Daniel Tamarit, Emelie Javelind, Tobias C Olofsson, Siv GE Andersson, Alejandra Vásquez

**Affiliations:** Department of Molecular Evolution, Cell and Molecular Biology, Science for Life Laboratory, Biomedical Centre, Uppsala University, Husargatan 3, SE-751 24 Uppsala, Sweden; Department of Laboratory Medicine, Medical Microbiology, Lund University, Medicon Village, Scheelevägen 2, SE-223 62 Lund, Sweden

**Keywords:** Lactic acid bacteria, *Lactobacillus* spp, Firmicutes, Bifidobacteria, Comparative genomics, Phosphotransferase systems, Niche specialization

## Abstract

**Background:**

In the honeybee *Apis mellifera*, the bacterial gut community is consistently colonized by eight distinct phylotypes of bacteria. Managed bee colonies are of considerable economic interest and it is therefore important to elucidate the diversity and role of this microbiota in the honeybee. In this study, we have sequenced the genomes of eleven strains of lactobacilli and bifidobacteria isolated from the honey crop of the honeybee *A. mellifera*.

**Results:**

Single gene phylogenies confirmed that the isolated strains represent the diversity of lactobacilli and bifidobacteria in the gut, as previously identified by 16S rRNA gene sequencing. Core genome phylogenies of the lactobacilli and bifidobacteria further indicated extensive divergence between strains classified as the same phylotype. Phylotype-specific protein families included unique surface proteins. Within phylotypes, we found a remarkably high level of gene content diversity. Carbohydrate metabolism and transport functions contributed up to 45% of the accessory genes, with some genomes having a higher content of genes encoding phosphotransferase systems for the uptake of carbohydrates than any previously sequenced genome. These genes were often located in highly variable genomic segments that also contained genes for enzymes involved in the degradation and modification of sugar residues. Strain-specific gene clusters for the biosynthesis of exopolysaccharides were identified in two phylotypes. The dynamics of these segments contrasted with low recombination frequencies and conserved gene order structures for the core genes. Hits for CRISPR spacers were almost exclusively found within phylotypes, suggesting that the phylotypes are associated with distinct phage populations.

**Conclusions:**

The honeybee gut microbiota has been described as consisting of a modest number of phylotypes; however, the genomes sequenced in the current study demonstrated a very high level of gene content diversity within all three described phylotypes of lactobacilli and bifidobacteria, particularly in terms of metabolic functions and surface structures, where many features were strain-specific. Together, these results indicate niche differentiation within phylotypes, suggesting that the honeybee gut microbiota is more complex than previously thought.

**Electronic supplementary material:**

The online version of this article (doi:10.1186/s12864-015-1476-6) contains supplementary material, which is available to authorized users.

## Background

Honeybees are social insects that divide labor and live in highly structured communities. As pollinators, the honeybees play an instrumental role in shaping natural ecosystems by facilitating gene flow between plants [1]. Furthermore, managed honeybee colonies provide pollination services for many agricultural crops, and are therefore of considerable economic importance [2]. Dramatic losses of honeybees in recent years have spurred research towards a better understanding of mutualistic and pathogenic microorganisms associated with the honeybee [3,4]. Some beekeepers use antibiotics to control pathogens, which in turn has affected the commensal microbiota and resulted in the accumulation of antibiotic resistances with unknown long-term consequences for honeybee health [5]. An improved understanding of the evolution and function of the honeybee microbiota is therefore an important step towards devising long-term viable management strategies for improving honeybee health.

Currently, little is known about the role of individual members of the commensal microbiota, and their interactions with each other and the honeybee host. However, similarly to the human gut microbiome [6-8], the honeybee microbiota is thought to be involved in the defense against pathogens and in the food processes within the beehive [9-11]. Several independent studies of samples from diverse geographic origins have shown that the healthy honeybee gut contains a specialized microbial community, dominated by eight distinct phylotypes [4,12-14]. Quantitative studies have indicated that the community composition fluctuates between honeybees and sites, but that the eight phylotypes generally represent >99% of all bacterial sequences in the gut metagenome of the worker bees [15-17].

Two phylotypes of the honeybee microbiota belong to the genus *Lactobacillus* of the phylum Firmicutes (named “Firm-4″ and “Firm-5″), with abundances in individual bees ranging from less than 5% to more than 50% [15-17]. A third phylotype belongs to the genus *Bifidobacterium* (named “Bifido”) of the phylum Actinobacteria. Similarly to the lactobacilli, bifidobacteria are consistently found in the honeybee gut microbiota, although at lower abundances [15-17].

Phylotypes, or species, are commonly inferred from a 97% cut-off in percentage identities for the 16S rRNA genes, under the assumption that strains in such groups are ecologically similar, but the adequacy of this cut-off is debated [18,19]. Notably, inconsistencies between the sequence similarity of the 16S rRNA genes and protein coding genes was recently reported for a single-cell genome study of the honeybee gut phylotypes *Gilliamella apicola* and *Snodgrasella alvi*, where it was suggested that recombination has homogenized the 16S rRNA genes within phylotypes, while other genomic regions have continued to diverge [20]. Similarly to *G. apicola* and *S. alvi*, a high similarity in the 16S rRNA genes for two strains of the “Firm-5″ phylotype contrasted with an average nucleotide identity of 86% for their genomes [21]. This suggests that the honeybee gut bacteria may be functionally divergent despite having highly similar 16S rRNA genes.

Honeybees are generalist pollinators and their main food sources are nectar and pollen produced by flowers. The sugar concentration in nectar varies widely, from less than 10% to more than 70% [22]. Nectar consists mainly of the disaccharide sucrose and its monosaccharides fructose and glucose, but the exact composition of sugars differs between continents, seasons and sources [22]. Consistent with the adaptation to a carbohydrate-rich diet, a metagenomic study of the bee gut identified protein families involved in carbohydrate metabolism and transport among the significantly enriched functional categories [23]. These include phosphotransferase systems (PTS) and enzymes involved in the breakdown of polysaccharides in nectar, pollen walls or host glycans, such as glycoside hydrolases, polysaccharide lyases and pectin.

Nectar collected from flowers is first stored in the crop, which is a highly osmotic and microaerophilic bacterial hostile environment that precede the mid- and hindgut. Despite the harsh conditions in the crop, bacteria have been isolated from this compartment, including *Lactobacillus kunkeii* [24] and diverse members of the “Firm-4″, “Firm-5″ and “Bifido” phylotypes described for the honeybee gut microbiota [10,25,26]. Thus, similar strains of *Lactobacillus* spp. and *Bifidobacterium* spp. have been isolated from the entire alimentary tract. The identified strains are found in all honeybees that belong to *Apis mellifera* and its subspecies regardless of the geographic location [10,27,28]. Previous research has demonstrated that the isolated bacterial strains secrete substances such as bacteriocins and antimicrobial proteins [29], and can inhibit the growth of the honeybee pathogens (*Paenibacillus larvae* and *Melissococcus plutonius*) and food spoilers *in vitro,* and *in vivo* in honeybee larvae [10,11,29]. However, at the genetic level, nothing is known about these strains beyond the 16S rRNA genes, and as we know from previous studies of other phylotypes of the honeybee gut microbiota, comparisons of the 16S rRNA genes may underestimate the divergence and diversity of the protein coding genes.

To study the correlation between the diversity of gene sequences and functions, we have sequenced and analyzed the genomes of 11 bacterial strains isolated from the crop of *A. mellifera.* The strains were selected to include representatives of the “Firm-4″, “Firm-5″ and “Bifido” phylotypes, several of which have recently been described as novel species [30]. By comparative genome analyses, including 6 recently published genomes of bifidobacteria isolated from honeybees and bumblebees [31,32], we have quantified sequence divergence levels, identified novel gene acquisitions and estimated recombination frequencies. We discuss the genome-wide level of diversity and the finding that each of the three phylotypes contains highly diverse communities of strains with distinct metabolic properties.

## Results

### Genome overview

We have sequenced the genomes of 11 strains of *Lactobacillus* and *Bifidobacterium* spp. (Table 1) isolated from the crop of *Apis mellifera mellifera*, as described previously [10,25,29]. For 9 of the 11 strains, most of the contigs could be organized into a single scaffold, and the number of contigs in the final assemblies ranged from 11 to 38 (Additional file 1: Table S1). Overall, the genomes showed the expected GC-skew curves that followed the putative origin and terminus of replication (Additional file 2: Figure S1A). Furthermore, read coverage generally displayed a smooth curve with a coverage peak around the origin and a valley at the terminus (Additional file 2: Figure S1B), suggesting that the genomes had been accurately assembled. Contigs not contained within the scaffolds included sequences from the rRNA regions. A highly increased coverage over these contigs indicated the presence of multiple rRNA gene copies. Most of the remaining contigs not included in the main scaffolds were putatively identified as plasmids.

**Table 1:** **Strains sequenced in the current study**

**Strain**	**Species** ^**1**^	**Genus**	**Bee gut phylotype** ^**2**^
Bma6	*B. coryneforme/B. indicum*	*Bifidobacterium*	Bifido-2
Bin2	*B. asteroides*	*Bifidobacterium*	Bifido-1
Bin7	*B. asteroides*	*Bididobacterium*	Bifido-1
Hma3	*B. asteroides*	*Bifidobacterium*	Bifido-1
Hma11	*L. apis*	*Lactobacillus*	Firm5
Bma5	*L. helsingborgensis*	*Lactobacillus*	Firm5
Hma8	*L. melliventris*	*Lactobacillus*	Firm5
Hma2	*L. kimbladii*	*Lactobacillus*	Firm5
Biut2	*L. kullabergensis*	*Lactobacillus*	Firm5
Hon2	*L. mellis*	*Lactobacillus*	Firm4
Bin4	*L. mellifer*	*Lactobacillus*	Firm4

^1^Species descriptions were recently published for the *Lactobacillus* strains in [30]. For the *Bifidobacterium* strains, the closest relative (based on the 16S rRNA gene) is indicated.

^2^The “Bifido” group was further divided into subgroups, based on the core genome phylogeny (Figure 2).

The genomes were about 2 Mb in size, ranging from 1.54 Mb (*L. apis*) to 2.13 Mb (*L. kimbladii*), and containing from 1,327 to 1,891 genes, of which from 44 to 258 genes (>300 nucleotides) showed no hits to genes in the public databases (Table 2). Plasmids were identified in 6 of the 11 strains, and some strains had more than one plasmid. For the bifidobacteria, two small plasmids were associated with strain Hma3, while none were found in the other strains, thus conforming to the general trend that only small plasmids are present in the *Bifidobacterium* genus [33]. In contrast, large plasmids of more than 100 kb were found in 4 of the *Lactobacillus* strains (Table 2). Prophage regions were putatively identified in most genomes. An increase in sequence coverage over the phage-regions was observed in the *L. kullabergensis* and *L. melliventris* genomes, indicating the presence of multiple phage gene copies or replication of the prophage. The latter is perhaps more likely since some read pairs supported circularization whereas other read pairs suggested that the region was located within the main chromosomal scaffold.

**Table 2:** **Descriptive statistics on genomes**

**Strain/species**	**Genome size** ^**1**^ **(bp)**	**Plasmids**	**Total plasmid size (bp)**	**Phage regions**	**GC content**	**# of CDS** ^**2**^	**Mean gene size (bp)** ^**3**^	**rRNA (5S, 16S, 23S)**	**tRNA**	**Pseudo-genes**	**Partial CDS** ^**4**^	**# Unique genes** ^**5**^
Bma6	1738065	None	NA	None	61	1327	1146	multiple	46	7	11	78
Bin2	2087605	None	NA	1	61	1569	1159	multiple	45	8	14	45
Bin7	2120336	None	NA	1	61	1576	1172	multiple	45	19	2	49
Hma3	2217062	2	15107	1	60	1672	1161	multiple	46	18	7	44
*L. apis*	1542091	1	137911	None	37	1498	964	multiple	49	14	10	180
*L. helsingborgensis*	1868619	1	123906	None	36	1730	986	multiple	49	20	18	198
*L. melliventris*	1956081	2	144786	1	36	1891	961	multiple	50	22	12	213
*L. kimbladii*	2130297	2	15247	1	36	1872	982	multiple	50	22	19	225
*L. kullabergensis*	2079016	None	NA	2	36	1844	975	multiple	50	21	21	199
*L. mellis*	1790038	None	NA	1	37	1572	976	multiple	53	18	7	258
*L. mellifer*	1681465	2	104625	2	39	1568	967	multiple	49	21	24	254

^1^Total size of contigs larger than 500 bp, excluding contigs annotated as plamids.

^2^CDS, excluding pseudogenes and partial genes.

^3^CDS (excluding pseudogenes and partial genes) divided by total length.

^4^CDS overlapping a contig border.

^5^Genes larger than 300 bp with no significant blast-hits found during annotation.

### Core phylogenies of lactobacilli and bifidobacteria

**Figure 1:**
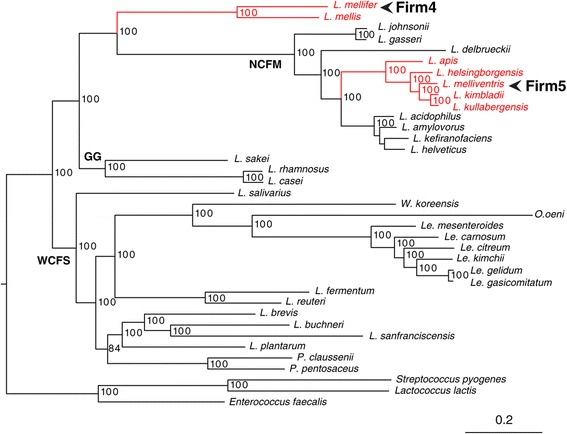
Core genome phylogeny of lactobacilli. Phylogenetic tree inferred from a maximum likelihood analysis of a concatenated alignment of 303 pan-orthologous genes. Strains sequenced in the current study are highlighted in red, with their group names indicated to the right (“Firm4” and “Firm5”). The main groupings of lactobacilli as identified by Kant *et al*. [34] are indicated with bold letters. Accession numbers of all genomes are listed in Additional file 3: Table S2 (L. = *Lactobacillus*, Le. = *Leuconostoc*, W. = *Weissella*, O. = *Oenococcus*, P. = *Pediococcus*).

To place the isolated strains in a phylogenetic context, we retrieved complete genome sequence data from all species of the families Lactobacillaceae and Leuconostocaceae (as of May 18, 2013) (Additional file 3: Table S2). We identified 6053 protein family clusters with Ortho-MCL for this set of genomes, of which 303 were single-copy genes present in all genomes. The sequences of the pan-orthologous proteins were aligned and concatenated, and used for a phylogenetic inference with the maximum likelihood method (Figure 1). This analysis showed that five of the *Lactobacillus* strains (*L. apis, L. helsingborgensis, L. melliventris, L. kimbladii, L. kullabergensis*) belonged to the so-called NCFM clade [34], named after *Lactobacillus acidophilus*. The two other *Lactobacillus* species sequenced here, *L. mellis* and *L. mellifer*, formed a separate strongly supported clade that diverged prior to the NCFM clade. Furthermore, the two species were not particularly closely related to each other (94% sequence similarity in the 16S rRNA gene), similarly to the diversity found in a recent study using the 16S rRNA gene [35].

**Figure 2:**
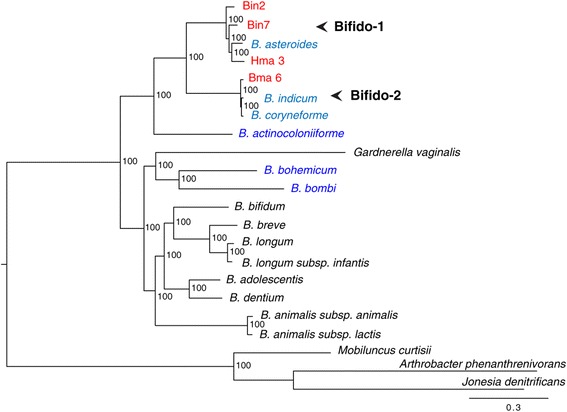
Core genome phylogeny of bifidobacterial strains. Phylogenetic tree inferred from a maximum likelihood analysis of a concatenated alignment of 400 pan-orthologous genes. Strains sequenced in the current study are highlighted in red, with their group names indicated to the right (“Bifido-1” and “Bifido-2”). Other strains isolated from the honeybee gut are shown in light blue, and strains isolated from the bumblebee gut are shown in dark blue. Accession numbers of all genomes are listed in Additional file 3: Table S2.

Likewise, we identified and aligned single-copy gene orthologs for each species of the genus *Bifidobacterium* for which complete genome data was available (as of May 18, 2013), to which we added five recently published genomes of bifidobacteria isolated from honeybees and bumblebees [31] (Additional file 3: Table S2) and the four genomes sequenced in the current study. We also included *Gardnerella vaginalis*, which is closely related to the genus *Bifidobacterium* and for which the taxonomic placement is debated [36]. We inferred a maximum likelihood phylogeny of these species based on the concatenated alignment of 400 single-copy orthologs (Figure 2). Strain Hma3, Bin2 and Bin7 formed a clade (“Bifido-1″) with *B. asteroides*, isolated from the gut of *Apis mellifera* [32,37], while strain Bma6 was part of a sister clade (“Bifido-2″) to this group, clustering together with *B. indicum* and *B. coryneforme*. The two “Bifido” groups and *B. actinocoloniiforme* (isolated from the bumblebee) formed a strongly supported clade that diverged prior to the common ancestor of previously sequenced *Bifidobacterium* species isolated from non-invertebrate habitats.

Notably, although these two groups of *Bifidobacterium* strains isolated from the honeybee are on average 98% identical in their 16S rRNA gene sequences (and as such are classified as the same phylotype), the lengths of the branches separating the groups indicate a higher level of divergence between the groups compared to previously sequenced bifidobacterial species with similar 16S rRNA divergences (i.e. 97-98% identity in 16S rRNA sequences between *B. breve* and *B. longum*). The two other strains isolated from the bumblebee (*B. bohemicum* and *B. bombi*) formed a separate clade with *G. vaginalis*, which also diverged prior to the common ancestor of previously sequenced bifidobacteria from non-invertebrate habitats. *G. vaginalis* is currently classified as the sole species of the genus *Gardnerella*, which in turn belongs to the Bifidobacteriaceae family. Thus, in the phylogeny presented here, the *Bifidobacterium* genus is paraphyletic suggesting that *Gardnerella* should be re-classified.

### Phylogenetic comparison to the honeybee gut microbiota

To examine the relationships of our strains to the phylotypes described for the digestive tract of *A. mellifera* [14], we also inferred phylogenetic trees based on the 16S rRNA and the *uvrC* genes. The 16S rRNA gene phylogeny of the *Lactobacillus* strains analyzed here and related sequences from the bee gut microbiota [12,14] confirmed that *L. mellifer* and *L. mellis* belong to the “Firm-4″ phylotype, and that *L. apis*, *L. helsingborgensis, L. melliventris, L. kimbladii* and *L. kullabergensis* belong to the “Firm-5″ phylotype (Additional file 4: Figure S2A, Table 1). Furthermore, the phylogeny inferred from the *uvrC* gene showed that each of the four “Firm-5″ phylotype *uvrC* sequences obtained from a metagenome sample of the honeybee gut [23] were more closely related to the species sequenced in the current study than to each other, indicating that these species are representative of the “Firm-5″ bacterial community in the honeybee (Additional file 4: Figure S2B).

Likewise, the diversity of the bifidobacterial strains from the crop matched the diversity of bifidobacterial sequences in the bee gut microbiota (Additional file 5: Figure S3). However, while the topologies of the 16S rRNA and *uvrC* gene phylogenies were largely consistent with the core genome phylogeny, the 16S rRNA gene phylogeny in particular was poorly supported, indicating that this genetic marker does not contain sufficient information for reliable phylogenetic inference within the genus *Bifidobacterium*. Based on the *uvrC* gene phylogeny, the two “Bifido” phylotype *uvrC* sequences from the honeybee gut microbiota [23] are most closely related to strain Hma3 in the current study, while strains Bin2 and Bin7 clustered with *B. asteroides* [37].

We conclude that the strains sequenced in the current study are representative of the *Lactobacillus* and *Bifidobacterium* phylotypes described for the honeybee gut microbiota. For reasons of consistency with the nomenclature used previously to describe the key members of the bee gut microbiota, we refer to the cultivated strains from the crop as the “Firm-4″, “Firm-5″ and “Bifido” groups in the following analyses, with “Bifido-1″ and “Bifido-2″ referring to each of the subgroups within the “Bifido” phylotype group (Figure 2, Table 1).

### Inference of the core and accessory genomes

The repeated isolation of multiple members of the same phylotype from the same apiary [25] suggests that the strains form stable communities, consistent with the simultaneous identification of most of the strains in an independent metagenomic dataset [23] (Additional file 4: Figure S2B, Additional file 5: Figure S3B). As a first step towards understanding the maintenance of these communities, we inferred the core and accessory genomes for each phylotype group.

**Figure 3:**
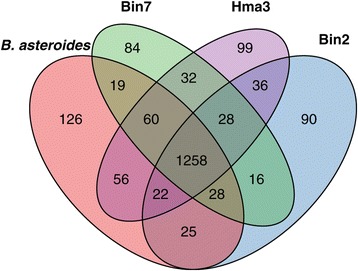
Venn diagram of shared protein families within the “Bifido-1” group. Numbers correspond to protein families of orthologous sequences, inferred with Ortho-MCL, plus singletons (proteins unique to a single strain). Similar plots for the “Firm-5” and “Bifido-2” groups can be found in Additional file 6: Figure S4 and Additional file 7: Figure S5.

From the two separate Ortho-MCL clusterings of the Lactobacillaceae/Leuconostocaceae and *Bifidobacterium* proteome datasets, we identified 1015 and 1046 single-copy orthologs present in all strains of the “Firm-5″ and “Bifido″ groups, respectively. To identify the accessory gene pools for each group, we counted protein family clusters (as estimated with Ortho-MCL) that lacked one or more strains for each group. In total, 1371 and 1076 protein families were found to be variably present within the “Firm-5″ and “Bifido” groups respectively. For the “Bifido” subgroups, 721 and 195 protein families were accessory in “Bifido-1″ and “Bifido-2″ respectively, out of which 37 were accessory in both. To visualize patterns of shared gene content within each of these groups, we plotted the accessory protein families, including singletons, in Venn diagrams (Figure 3, Additional file 6: Figure S4 and Additional file 7: Figure S5). These plots revealed an approximately even distribution of shared protein families, with protein families being shared between strains in all possible combinations, consistent with ongoing gene losses and/or horizontal gene transfers. For the “Firm-4″ group, which was only represented by two strains in the current study, the core and accessory genomes were not estimated. However, we noted that 373 and 382 protein family clusters/singletons did not include both strains. Thus the sequenced genomes suggest a very high level of gene content diversity within all three groups.

**Figure 4:**
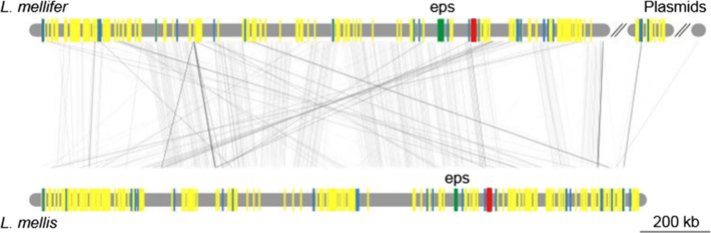
Genome synteny plot of the “Firm-4″ strains. Comparative analysis of the “Firm-4” genomes. The genome and plasmid sequences are represented by horizontal grey lines. The similarity between genomes was inferred with blastn and is shown with connecting grey lines, where darker lines indicate higher similarity. Blue bars show the positions of conserved group-specific core genes. Yellow bars indicate the positions of genes, which are not shared between the two strains. Red bars indicate the conserved group-specific operon encoding the putative *cscAB* genes [52], whereas green bars show the position of putative *eps*-clusters.

**Figure 5:**
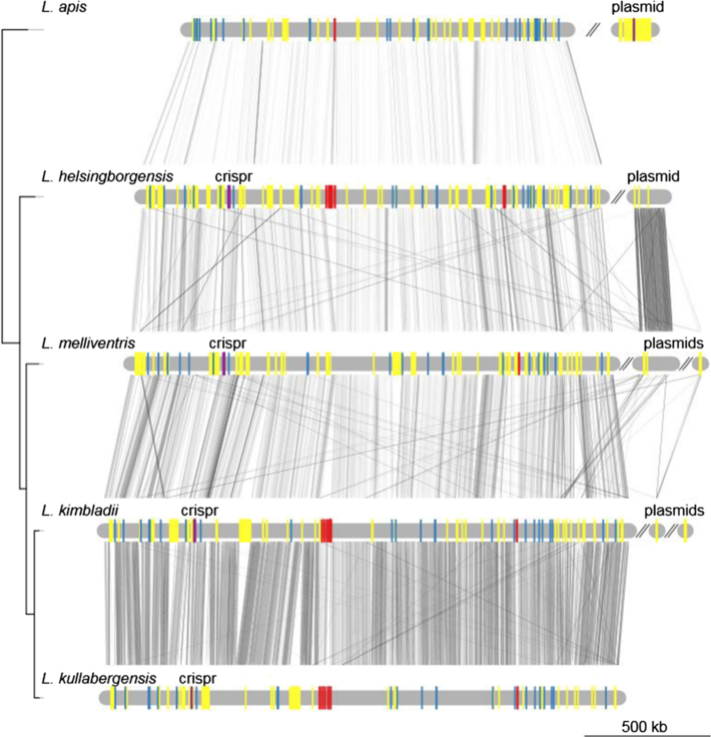
Genome synteny plot of the “Firm-5” strains. Comparative analyses of the “Firm-5” genomes. The genome and plasmid sequences are represented by horizontal grey lines. The similarity between genomes was inferred with blastn and is shown with connecting grey lines, where darker lines indicate higher similarity. Blue bars show the positions of conserved group-specific core genes. Yellow bars indicate the positions of genes, which are strain-specific. The positions of the putative surface-exposed proteins are indicated in red. CRISPR genes are shown in purple. The tree topology is as in Figure 1.

**Figure 6:**
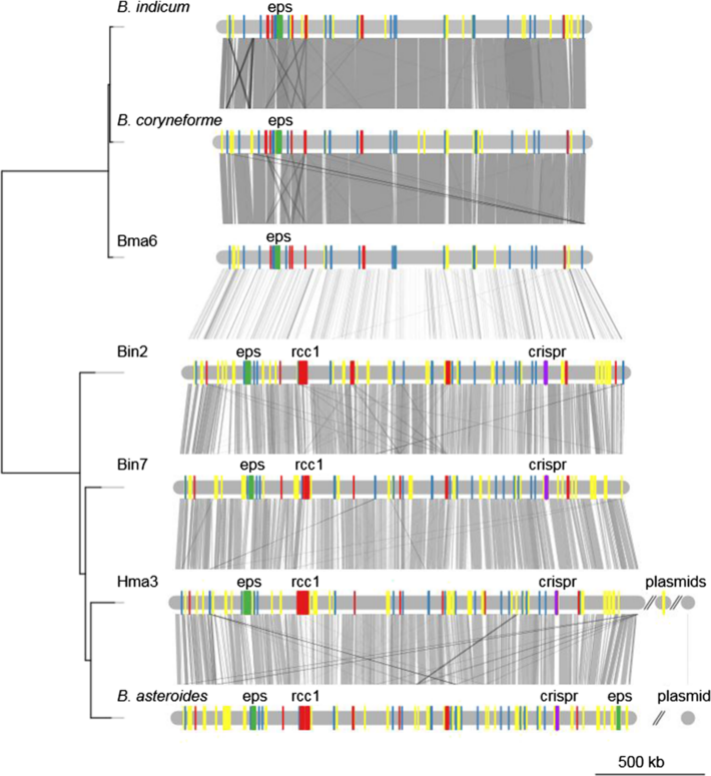
Genome synteny plot of the “Bifido” strains. Comparative analyses of the “Bifido” genomes. The genome and plasmid sequences are represented by horizontal grey lines. The similarity between genomes was inferred with blastn and is shown with connecting grey lines, where darker lines indicate higher similarity. Blue bars show the positions of genes conserved within the “Bifido-1” and “Bifido-2” groups respectively. Yellow bars indicate the positions of genes, which are strain-specific. Red bars indicate the positions of genes containing the RCC1-repeat domain, where the largest region is indicated with text. CRISPR genes are shown in purple. Green bars show the positions of putative *eps*-clusters. The tree topology is as in Figure 2.

Despite the presence of large accessory gene pools, the gene order of the genomes was highly conserved within the groups, with strain-specific genes scattered along backbones of conserved core genes (Figures 4, 5 and 6).

### Adaptation to the arthropod and mammalian gut

The honeybee and bumblebee digestive tract, including crop, mid- and hindgut, represents a unique environment compared to previously described habitats for *Lactobacillus* and *Bifidobacterium* species. Therefore, core genes specific to strains isolated from bees are candidates for traits associated with adaptation to the arthropod gut. We identified about 20–40 protein families uniquely present in each of the “Firm-4″, “Firm-5″ and “Bifido” groups, but not in the proteomes of their related species in Lactobacillaceae/Leuconostocaceae and *Bifidobacterium*. Although most of these proteins were of unknown function, several interesting group-specific gene functions were identified.

Among the genes shared exclusively among the *Bifidobacterium* strains isolated from bees were the *cydABCD* genes involved in aerobic respiration (previously described for *B. asteroides* [32]), suggesting that these genes are important for colonization of the arthropod gut for bifidobacteria. The *cydABCD* genes were also present in the “Firm-4″ group, but not in the “Firm-5″ group, possibly reflecting adaptation to distinct microhabitats within the gut. The c*ydABCD* genes have previously been shown to be sporadically present among the lactobacilli, with several independent gene losses being the most parsimonious explanation for their scattered distribution pattern [38]. Consistently, the GC content of the *cydABCD* genes of the “Bifido” genomes was similar to the genomic GC content, suggesting that this gene cluster was ancestrally present and has been lost at the node separating the bee-associated bifidobacteria from the other bifidobacterial genomes.

The group-specific genes also included genes coding for compounds involved in carbohydrate storage. Trehalose is used for carbohydrate storage in bacteria, yeast and insects [39], whereas glycogen is the main carbohydrate storage compound in mammals. Uniquely present in the bee-associated bifidobacteria were the *otsAB* genes, which code for enzymes involved in the biosynthesis of trehalose. In contrast, the genes for glycogen biosynthesis (*glgABC*) and degradation (*glgPX*) were absent in the bee-associated bifidobacteria, although these were otherwise conserved in all other bifidobacterial genomes included in this study. Likewise, homologs of the glycogen biosynthesis operon in *Lactobacillus acidophilus* [40] could not be detected in either of the “Firm-4” and “Firm-5” groups.

### Novel group-specific outer surface proteins of the honeybee gut microbiota

**Figure 7:**
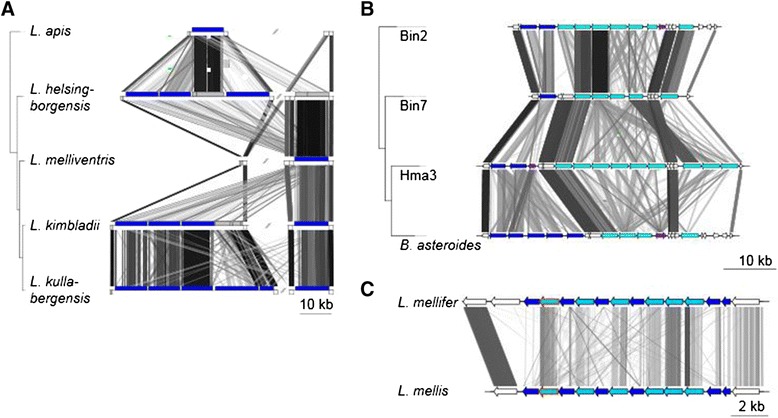
Comparative analysis of regions containing group-specific putative outer-surface proteins. A) Two genomic regions containing duplicated genes for novel putative surface exposed proteins in “Firm-5” strains. Genes are shown as boxes, where blue and grey boxes represent the putative outer surface proteins, and white boxes represent other genes in the regions. Grey boxes correspond to genes identified at contig borders in the genome assemblies (partially assembled). The tree topology is as in Figure 1. **B**) Comparative analysis of the main genomic region containing tandemly duplicated genes for RCC1-repeat domain proteins in “Bifido” strains.” Genes are shown as arrows, where genes corresponding to each of the two protein clusters indicated by OrthoMCL and phylogeny (Additional file 8: Figure S6) are shown in light and dark blue respectively. Genes containing RCC1-repeats that are members of other protein familes, as predicted by OrthoMCL, are shown in purple. Genes not containing the RCC1-repeat domains are shown in white. The genomic position of the region is indicated in Figure 6. The tree topology is as in Figure 2. **C**) Genomic regions containing the putative *cscAB* genes in “Firm-4″ strains. Genes are shown as arrows, where light-blue arrows represent the putative *cscA* genes, and dark-blue arrows represent the putative *cscB* genes. The gene with homologues in other lactobacilli strains used for the OrthoMCL search is shown with a red border. In all comparisons, the similarity between genomes was inferred with tblastx and is shown with connecting grey lines, where darker lines indicate higher similarity.

Several of the core genes specific to the “Firm-4″, “Firm-5″ and “Bifido″ groups were inferred to code for outer surface structures based on protein domain predictions. The “Firm-5″ group contained a variable number of tandemly repeated genes coding for large proteins of 1500 to 4600 amino acids found in two distant genomic locations (Figure 5, Figure 7A). Some of the genes were located at assembly contig borders (shown with grey boxes in Figure 7A), suggesting that additional copies may be present in some strains. The proteins were all predicted to contain an YSIRK signal motif in the N-terminal segment, up to five copies of a domain of unknown function (DUF1542) in the central part of the protein, and two SLAP domains at the C-terminal end. The YSIRK motif serves as a signal peptide for protein secretion in *Staphylococcus* and *Streptococcus* [41], and has been identified in various *Lactobacillus* adhesins [42-45]. The SLAP domain is the common denominator for surface layer (S-layer) proteins in the NCFM clade [46], and mediates binding of the S-layer protein to the cell envelope [47-50]. However, while the previously identified *Lactobacillus* S-layer proteins are in the size range of 40–200 kDa and encode a single SLAP domain, these group-specific putatively surface-exposed proteins of the “Firm-5″ clade are substantially larger, 350–500 kDa, and encode two SLAP domains.

The “Bifido” genomes contained 7–24 genes per genome coding for RCC1 repeat domain proteins, previously identified in 19 gene copies in the *B. asteroides* genome [32]. Some additional RCC1-repeat domain proteins were found at assembly contig borders (partially assembled genes), likely due to the repetitive nature of the proteins. Furthermore, 10 RCC1-repeat domain proteins were present in *B. actinocoloniiforme*, the most closely related outgroup strain to the “Bifido” group (Figure 2), suggesting the genes were acquired in the common ancestor of the clade, and have since diversified. Most of the RCC1 repeat domain proteins were co-located in clusters with tandemly repeated genes (Figure 6, marked in red), the longest cluster of which is shown in Figure 7B. All genes were found to encode a *Listeria-Bacteroides* repeat domain (Flg_new) upstream of each RCC1-domain, and the cell wall anchoring signal LPxTG sequence at the C-terminal end with a hydrophobic stretch of amino acids and a short positively charged tail [51], indicating that the RCC1-domain proteins are covalently attached to the peptidoglycan.

On average, we identified 7 repeats per domain, with 2–4 domains per protein. Interestingly, the two “Bifido” subgroups differed in both the number and type of RCC1-repeat domain proteins encoded in the genomes. The RCC1-repeat domain proteins of the “Bifido-1″ subgroup mainly contained 2 domains, with a single 3-domain protein present in all “Bifido-1″ strains and no 4-domain proteins. In contrast, the “Bifido-2″ subgroup strains had multiple RCC1-repeat domain proteins with 3 domains as well as a single 4-domain protein conserved in all strains. Phylogenetic inference based on the RCC1-repeat domain proteins with two (Additional file 8: Figure S6) and three domains respectively (Additional file 9: Figure S7) also revealed a clear separation of the subgroups, with the *B. actinocoloniiforme* RCC1 repeat-domain proteins forming a separate clade from the two subgroups. In contrast, the clustering of proteins from the same subgroup was highly variable, and the proteins were generally not positional orthologs. We conclude that the RCC1 repeat domain proteins evolve by duplication and divergence under diversifying selection, with recombination and horizontal gene transfers mainly restricted to the subgroup level.

The “Firm-4″ genomes contained 11 conserved group-specific protein families residing in a contiguous region of 12 genes (Figure 4, Figure 7C). Six of these genes code for proteins with two domains of unknown functions (DUF916 and DUF3324), and three for a protein containing the WxL domain. *L. mellifer* also contained one additional gene outside the cluster coding for a protein with the WxL domain. These domains have previously been identified in several plant-associated gram-positive bacteria, and were found to be particularly numerous in *Lactobacillus plantarum* [52]. Genes containing these domains are organized into nine clusters in *L. plantarum,* each of which contains one or more copies of the *cscABCD* genes, where *cscA* code for proteins with the DUF916 domain and *cscBC* for proteins that contain the WxL domain [52]. The functional role of WxL domain proteins has not been determined, but the domain has been demonstrated to bind to the cell wall of gram-positive bacteria [53], and to mediate co-aggregation with other bacteria [54]. Additionally, it has been proposed that the *cscABCD* genes in *L. plantarum* encode cell-surface protein complexes involved in the degradation and utilization of complex plant polysaccharides [52]. Positional orthologs formed separate clusters with OrthoMCL, suggesting that the duplications arose before the separation of the *L. mellifer* and *L. mellis* species.

### Carbohydrate metabolism and transport functions dominate the accessory gene pool

The maintenance of a diverse bacterial community consisting of phylotypes with large accessory gene contents is suggestive of niche differentiation within the phylotypes. In order to gain clues about such potential differentiation, we functionally characterized the accessory genes. About 40-50% of the accessory protein families in the “Firm-5″ and “Bifido” groups could be assigned to a COG category. Among these, the COG category “carbohydrate metabolism and transport” was highly over-represented. Overall, 100–250 protein families per strain were assigned to carbohydrate transport and metabolism, of which about 60 were conserved among all strains of each phylotype. This represents 21–43% of the variably present families with a COG annotation, as compared to only 9-17% of the total proteome.

### Expansion of phosphotransferase systems in the “Firm-5″ group

In the “Firm-5″ group, the accessory gene pool was dominated by PTS transporters, which represented 50-60% of the 40–180 accessory genes assigned to COG category “G” in each strain. We assigned the identified transporters to the 7 described PTS transporter families with the aid of the transporter classification database [55] (see methods) and found that the large majority of genes coded for transporters of the Mannose-Fructose-Sorbose (Man) family (4.A.6) (Additional file 10: Figure S8). *L. kullabergensis* and *L. kimbladii* contained as many as 69 and 73 genes per genome for the Man transporter family, corresponding to at least 15 and 16 complete transporter operons with genes for all four subunits respectively.

**Figure 8:**
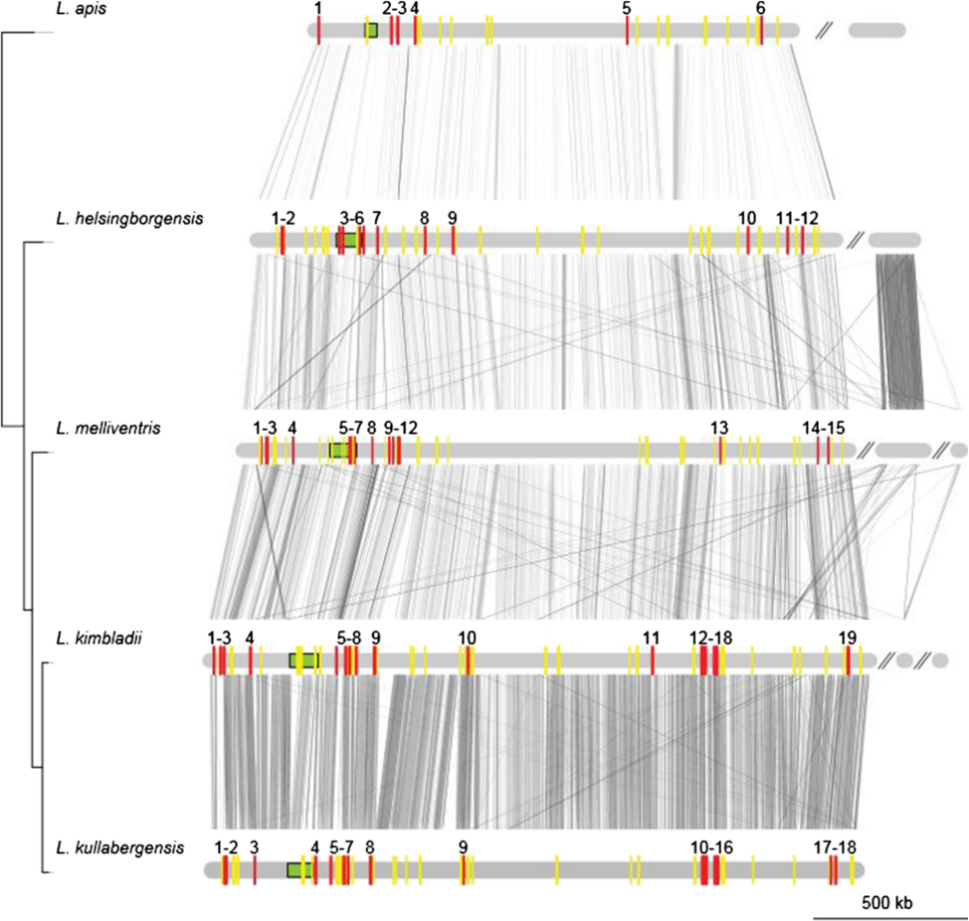
Genomic locations of PTS transporters in the genomes of the “Firm-5” strains. Genome sequences, similarity and phylogeny are as for Figure 5. PTS transporters are shown as red and yellow bars along the genomes, where red bars represent the Man family PTS transporters and yellow bars represent all other PTS transporters. Additionally, the Man PTS transporters have been numbered according to the order of their occurrence in the genomes. The green boxes show the position of the variable region analyzed in Figure 9.

**Figure 9:**
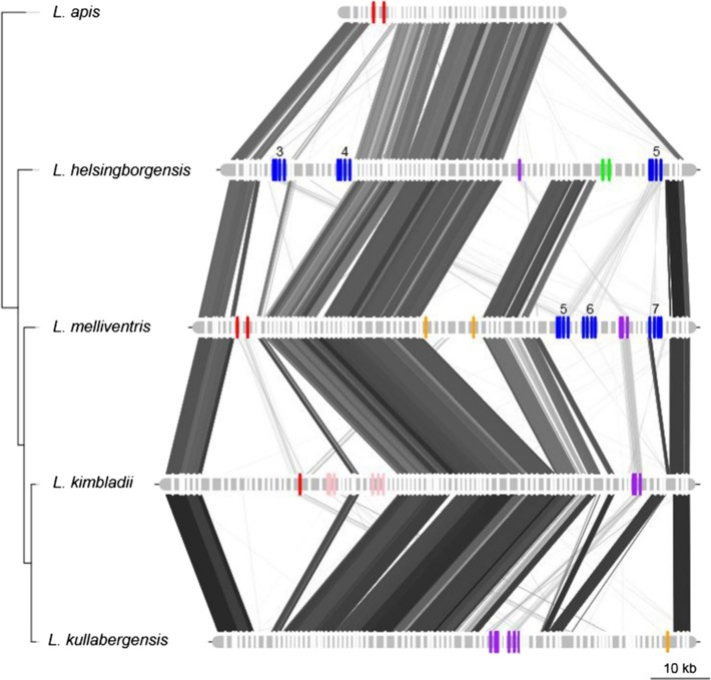
Hyper-variable regions containing multiple diverse PTS transporters. Genes are shown with bars along the sequences, where PTS transporters are shown in colour, and all other genes are shown in white. Red = Glc family, blue = Man family, purple = Gat family, green = Fru family, orange = Lac family, pink = Asc family (family designation according to [55]). Numbers above Man family transporters indicate their annotations as shown in Figure 8. The strain phylogeny is as in Figure 1, and similarity between the sequences is shown with connecting grey lines as estimated using tblastx with no filtering.

Although the PTS transporters were not restricted to any specific part of the genomes, different PTS transporters were often found in genomic islands with a general lack of sequence similarity between genomes (Figure 8). A more detailed plot of one such region with multiple different PTS transporters is shown in Figure 9. This region contains several genomic islands with variable numbers and families of PTS transporters. Not even the most closely related species, *L. kimbladii* and *L. kullabergensis*, have a similar set of genes for PTS transporters in the same order in this region.

To investigate the evolutionary relationships of the PTS genes in more detail, we inferred phylogenies of the Man PTS transporters based on the Man IIC and Man IID protein subunits (Additional file 11: Figure S9). The topology provided statistical support for more than 20 different groups, only one of which contained positional orthologs in all strains. In the two most closely related species *L. kimbladii* and *L. kullabergensis* 18–19 PTS transporter operons were identified, of which 13 sites were orthologous. Additional Man PTS gene clusters in *L. kullabergensis* were most similar to Man PTS genes in *L. melliventris*, while one showed a more distant relationship to Man PTS genes in *L. mellis* of the “Firm-4″ clade. Overall, these results suggest that the PTS transporters have undergone an extreme expansion, which preceded the diversification of the “Firm-5″ strains, followed by loss, recombination, diversification and possibly also transfer between the “Firm-4″ and “Firm-5″ groups.

Furthermore, we found that the PTS genes were co-located with other genes also involved in carbohydrate metabolism, including enzymes involved in the degradation and modification of sugar residues, such as glucosidases, hydrolases, isomerases, racemases, epimerases, aldolases and phosphatases, and their regulatory genes. Thus, the genomic islands in the “Firm-5″ group code mainly for carbohydrate-related functions and are extremely dynamic in structure and gene content.

### Diversity of the *eps* clusters within the “Firm-4″ and “Bifido” groups

**Figure 10:**
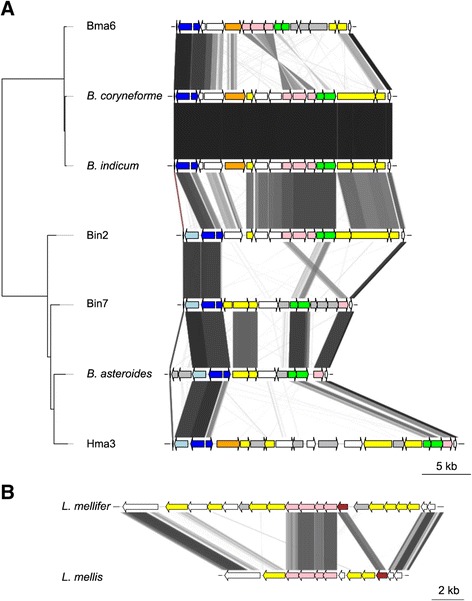
Comparative analysis of putative *eps*-clusters. **A**) Putative *eps*-clusters for the “Bifido” strains. Phylogenetic tree is as in Figure 2. **B**) Putative *eps*-clusters for the “Firm-4″ strains. Similarity was estimated with tblastx, using a length filter of 100 bp. Pink: dTDP-rhamnose biosynthesis genes, Green: ABC transporter genes, Yellow: glycosyl-transferases or genes with orthology to known *eps* genes, Orange: glycosyl-hydrolases, Brown: C-terminal domain of priming glycosyl-transferase, Grey: other genes with a putative function in polysaccharide biosynthesis. Light blue: putative catalase, dark blue: putative manganese transporter and repressor. For a complete list of protein domain predictions, see Additional file 12: Table S3.

Within the accessory gene content of the “Bifido” group, we identified carbohydrate ABC transporters as well as many enzymes involved in the degradation of complex carbohydrates, such as xylan and mannan (which are plant cell wall components), starch, and cellulose. We also identified a hyper-variable region coding for accessory proteins with a putative function in the biosynthesis of cell wall associated polysaccharides (Figure 10A). This region contains a gene cluster for the biosynthesis of the rhamnose precursor “dTDP-rhamnose” in the “Bifido-2″ group as well as in the “Bifido-1″ strain Bin2 (*rfbA,* fused *rfbCD* and *rfbB*). In the other “Bifido-1″ strains, only the *rfbB* gene could be identified.

In bifidobacteria, genes for the biosynthesis of dTDP-rhamnose are located within the *eps* gene cluster for the biosynthesis of exopolysaccharides (EPS) [56]. Likewise, it has been shown that dTDP-rhamnose is an important precursor of cell wall polysaccharides and rhamnose-containing EPS in *Lactococcus lactis* [57]. Two ABC-2 type transporter genes were also present in the hyper-variable region, which may be involved in the transfer of polysaccharides across the cytoplasmic membrane to the cell wall. Multiple genes for diverse glycosyl-transferases were present in all strains, consistent with a function in EPS biosynthesis [58], as well as other proteins with domains suggesting a function in polysaccharide biosynthesis (Additional file 12: Table S3). Finally, the GC-content of the genes in the region varied between 53-55%, compared to the genomic average of 60-61%, which is typical of *eps* clusters in bifidobacteria [56].

Since the segment containing the *eps* genes is located at the same genomic position in all genomes (Figure 6), we were able to identify the corresponding genes in the bumblebee-associated outgroup strain *B. actinocoloniiforme* (Additional file 13: Figure S10). The order of genes was similar to the “Bifido-1″ strain Bin7, but also included genes for a putative cell wall hydrolase present in all the “Bifido-2″ strains and the “Bifido-1″ strain Hma3. Thus, similarly to the diversity of PTS transporters in the genomic islands of the “Firm-5″ group, all the “Bifido” genomes contain unique combinations of genes within the *eps*-region (except for the two very closely related strains *B. indicum* and *B. coryneforme*) (Figure 10A), suggesting that the strains produce distinct cell wall associated polysaccharides. Furthermore, an *eps* cluster located in another genomic island was previously identified in *B. asteroides* [56], but was found to be absent from the “Bifido” strains sequenced in the current study (Figure 6).

No *eps*-clusters were identified within the “Firm-5″ group, but *L. mellifer* and *L. mellis* contained the *rfbABCD* genes for the biosynthesis of dTDP-rhamnose (Figure 10B). Furthermore, a gene encoding the C-terminal domain of the “priming” glycosyl-transferase, which is predicted to carry out the transferase function of the gene, was identified in both species. Similarly to the putative *eps*-cluster identified for the “Bifido” group, the two strains contained a unique combination of genes in the region, including multiple glycosyl-transferases, as well as genes with homology to the plasmid-encoded *eps*-cluster of *Lactococcus lactis* [59,60].

### Carbohydrate fermentation patterns

In an effort to evaluate the metabolic capacity of the strains, we determined the carbohydrate utilization profiles of the *Bifidobacterium* strains sequenced in the current study (Additional file 14: Figure S11), and compared these to the profiles of the *Lactobacillus* species [30]. All strains were able to grow on fructose and glucose, and these were the sole carbon sources that promoted growth of the “Firm-4″ strains among the sugars tested. Additionally, four of the “Firm-5″ strains possessed the ability to ferment mannose. The “Bifido” strains showed the broadest spectrum of metabolic capacities among the tested sugars; all strains utilized saccharose and arabinose, and an additional six sugars were fermented by one or more strains.

### Low levels of homologous recombination in the core genome

To place the dynamic changes of the genomic islands in the context of the core genome, we inferred individual gene phylogenies and compared their topologies to the topology of the concatenated phylogenetic trees (Figures 1 and 2). The “Firm-5″ and “Bifido” groups were in most cases monophyletic, suggesting that recombination events outside the groups that span across the gene boundaries are rare. Furthermore, out of 1046 single gene phylogenies, only two ABC-transporter genes and the protein encoded by the *rfbB* gene in *eps*-cluster of the “Bifido” group (Figure 10A) were incongruent with the monophyly of the “Bifido-1″ and “Bifido-2” groups.

To estimate the extent of shorter recombination tracts within genes, we used the two larger core gene datasets consisting of 1015 and 1046 single-copy genes present in all members of the "Firm-5″ and “Bifido″ groups, respectively. We applied three recombination-scanning methods in PhiPack (NSS, MaxChi and Phi) on each alignment and found 89 genes with evidence of recombination in the “Firm-5″ group (8.7%), while only 19 genes were significant for recombination in the “Bifido” group (1.8%).

Finally, we quantified the overall ratio at which recombination and mutation events (*r/m*) have generated substitutions in the strains sequenced here. To this end, ClonalFrame was run on genes previously used in multilocus sequence typing of *Lactobacillus casei* (*fusA, ileS, leuS, pyrG, recA* and *recG*) and *Bifidobacterium* spp. (*fusA, ileS, gyrB, rplB* and *rpoB*). The *r/m* ratio was estimated for these gene sets to 1.84 for the “Firm-5″ group (95% credibility region, 1.11-3.21) and to 0.60 for the “Bifido” group (95% credibility region, 0.26-1.06).

### Phylotype-specific diversity in adaptive immunity against phages

Phages and plasmids were inferred to be present in most of the genomes sequenced in the current study (Table 2). Defense CRISPR-*cas* systems [61] were identified in members of “Firm-5″ and the “Bifido-1” groups, but not in the “Firm-4″ or the “Bifido-2″ groups (Table 3). The “Firm-5″ strains encoded a CRISPR-cas system of type II-A, located at the exact same site in all genomes (Figure 5, Additional file 15: Figure S12A), while the “Bifido-2″ subgroup encoded CRISPR-cas systems of type I-E and I-C, also located at the same site in all genomes (Figure 6, Additional file 15: Figure S12B).

**Table 3:** **CRISPR-cas systems** ^**1**^

**Group**	**Species/strain**	**CRISPR classification** ^**1**^	**Number of spacers per region**
“Bifido-1”	Bin2	I-E	94
	Bin7	I-E	81
	Hma3	I-C^2^	63
	*B. asteroides*	I-E	146
“Bifido-2”	Bma6	NA	NA
	*B. coryneforme*	NA	NA
	*B. indicum*	NA	NA
“Firm-5”	*L. apis*	Incomplete^3^	5
	*L. helsingborgensis* (region 1)	II-A	9
	*L. helsingborgensis* (region 2)	Incomplete^4^	9
	*L. melliventris*	II-A	32
	*L. kimbladii*	II-A	91
	*L. kullagergensis*	II-A^5^	20
“Firm-4”	*L. mellis*	NA	NA
	*L. mellifer*	NA	NA
NA^6^	*B. actinocoloniiforme*	I-E	80

^1^Classified according to [101].

^2^Frameshift in gene *Cas3.*

^3^ *cas9* gene present on plasmid, fragment of *cas9* gene on chromosome.

^4^No *cas* genes, repeats are identical to region 1, but the spacers are different.

^5^Frameshift in gene *csn2.*

^6^ *B. actinocoloniiforme* is a *Bifidobacterium* species closely related to the “Bifido” phylotype strains, which was isolated from the bumblebee.

In the “Firm-5″ group, a second region of CRISPR spacers was found for *L. helsingborgensis* in another location on the chromosome, but without any associated *cas-*genes. The earliest diverging species, *L. apis*, contained only the degenerate first part of the *cas9* gene on the chromosome, but a complete *cas9* gene was found on its plasmid together with a few CRISPR spacers (Additional file 15: Figure S12A), suggesting that plasmids can mediate exchange of CRISPRs between strains.

The outgroup species of “Bifido-1″ and “Bifido-2″, *B. actinocoloniiforme*, encoded a type I-E CRISPR-cas system. Located upstream of these genes was a fatty acid biosynthesis operon, including the multifunctional type I fatty acid synthase gene (*fas*) (Additional file 15: Figure S12B). Surprisingly, the “Bifido-2″ strains lacked both the CRISPR-cas genes and the fatty acid biosynthesis operon, which was otherwise present in all bifidobacteria analyzed in the current study. A blast using the type II fatty acid biosynthesis gene *fabF* from *Arthrobacter phenanthrenivorans* (which was used as outgroup strain, Figure 2) did not yield any significant results in the “Bifido-2″ group, nor could any fatty acid biosynthesis genes be predicted using KAAS [62], so it is currently unclear how these strains synthesize fatty acids.

Next, we investigated whether the spacers of the identified CRISPR-cas systems (from honeybee and bumblebee associated strains) had similarity to any known sequences, or to sequences contained within the genomes analyzed in the current study. In total, hits were found for 26 out of 781 spacers (Additional file 16: Table S4). Most of the hits were to genes with a putative phage function, or hypothetical genes close to phage-related genes, and many hits were targeting the same genomic regions. All the spacers from the “Firm-5″ group for which a hit could be found were targeting other members of the “Firm-5″ group. Similarly, spacers originating from bifidobacteria isolated from honeybees or bumblebees had hits to other members of this group, with the exception of *B. bohemicum*, for which two spacers had hits to *B. longum*. Furthermore, one spacer from the recently sequenced genome of *Gilliamella apicola* had a hit to the identified plasmid from the “Firm-5″ species *L. apis*, albeit with multiple mismatches. These results suggest that strains of different phylotypes are subject to distinct phage populations within their shared habitat.

## Discussion

In this study, we have sequenced and analyzed 11 genome sequences of *Lactobacillus* and *Bifidobacterium* spp. isolated from the crop of the honeybee. We have shown that the strains represent the diversity of *rrs* and *uvrC* genotypes of the ″Firm-4″, “Firm-5″ and “Bifido” phylotypes, previously identified in the gut [14,26], and therefore consider our dataset to be a good representation of these phylotypes in the honeybee. Notably, the genomes revealed extensive diversity in gene content. In the following, we will discuss these results in light of adaptation and niche differentiation, both within and between the *Lactobacillus* and *Bifidobacterium* phylotypes associated with honeybees and bumblebees.

### Niche differentiation between phylotypes

As seen from the concatenated protein phylogenies, each of the three gram-positive phylotypes investigated in this study are more closely related to bacteria isolated from other habitats than to each other, and have therefore adapted to the honeybee independently, as already suggested for lactobacilli based on 16S rRNA gene sequences [63]. Consistently, we identified several protein families and functions specific to the bee-associated strains, but not shared between phylotypes. For example, genes for aerobic respiration were present in the “Bifido” and “Firm-4″ groups, but not in the “Firm-5″ group. Novel outer surface protein families, unique for each group were also identified, which are likely to be involved in interactions with both the host and the environment [41,46,64]. Furthermore, transporters and enzymes involved in import and degradation of sugar compounds differed extensively between the phylotypes. Taken together, this suggests that the three groups not only have different origins but also occupy distinct micro-habitats within the bee gut.

Similarly to the human gut microbiome, it has been proposed that the bee gut microbiota form a nutritional symbiotic association where the gut bacteria metabolize nutrients that the host cannot process. Indeed, a recent study showed that the honeybee gut microbiota is capable of metabolizing diverse compounds [65]. While nectar mainly consists of sucrose, glucose and fructose, trace amounts of other carbohydrates are also present, some of which are poisonous to the honeybees [66,67]. Thus, it was previously suggested that the “Firm-5″ strains could be responsible for processing mannose, based on the high diversity of PTS-transporters of the mannose family in a metagenomic sample [23]. Our genome analysis confirmed the presence of an exceptionally large number of PTS-transporters for the “Firm-5″ group and four of the five species within this group have been shown to be able to ferment mannose *in vitro* [30]. However, mannose was also fermented by two “Bifido” strains in the current study, so the fermentation of mannose is not strictly phylotype-specific.

In terms of general patterns of adaptation to the gut environment, it has previously been suggested that the biosynthesis of glycogen, and its use for carbohydrate storage, represents a specific adaptation in lactobacilli to the mammalian gastrointestinal tract [40]. Interestingly, we found that these biosynthetic genes were absent from the “Bifido”, “Firm-4″ and “Firm-5″ strains isolated from bees, which provides indirect support for the hypothesis that glycogen biosynthesis is indeed a specific adaptation to the mammalian gut. Instead, we identified genes for the biosynthesis of trehalose, which functions as an energy storage compound in insects. In bees, trehalose is produced in the fat body and maintained at high concentrations in the haemolymph [68]. Although more data is needed, it is intriguing that the gut bacteria seem to utilize the same storage compounds as the hosts to which they are adapted.

However, several other functions for trehalose have also been described in bacteria, such as stabilization of proteins and membranes during various stress-conditions, and protection from damage by oxygen radicals [39]. Considering the concomitant presence of the respiratory chain complex (*cydABCD*) in all the bee-associated bifidobacteria, it is possible that trehalose helps protect against oxidative stress. Although a number of other candidate genes were previously proposed to serve this function [32], none of these were conserved in all strains associated with honeybee and bumblebee associated strains in this study. However, an argument against such a general role is that no orthologs of the trehalose biosynthetic genes could be identified in the “Firm-4″ group, which also encodes the *cydABCD* operon.

### Niche differentiation within phylotypes

Our study has shown that about 40-50% of the identified genes in the genomes are variably present among strains of the same phylotype. Interestingly, phylotype sequences of the “Firm-5″ group from the same metagenomic study were more similar to the species sequenced in the current study than to each other, suggesting that these species are maintained in the honeybee colony. This result is particularly remarkable when considering that the protocol used for DNA extraction in the metagenomic study was designed to enrich for gram-negative bacteria, and therefore represents a conservative estimate of the diversity of gram-positive bacteria [23]. Although the basis of the co-existence of these species in the honeybee is not known, niche differentiation connected to the phylotype accessory gene content is an intriguing possibility.

Within the “Firm-5” group, an exceptionally large number of PTS transporters were identified; for example, *L. kullabergensis* and *L. kimbladii* from the “Firm-5″ group encode an estimated 41 and 42 complete PTS transporters, a diversity that to our knowledge is unprecedented [69,70]. For comparison, *L. plantarum* contains 25 complete operons for PTS transporters, and this has until now represented the largest number of PTS transporters in lactobacilli genomes [71]. While other members of the NCFM clade, to which the “Firm-5″ group belongs, also encode multiple PTS transporters, none of them encode as many as the “Firm-5″ strains [69,71]. Thus, the evolution of the “Firm-5″ group has likely been driven by selection for expansion and loss of the PTS transporters. Consistently, the PTS genes were mostly located inside genomic islands containing strain-specific sets of PTS genes, indicative of high rates of duplications, losses and gains. Similarly, a previous evolutionary study of Man family PTS transporters also showed that the transporter phylogenies were generally incongruent with the species phylogeny [70].

Despite the name, PTS transporters of the Man family are also known to import other sugars than mannose, and individual transporters may import a range of sugars [72]. Considering the extensive diversity of sequences within the Man transporter genes in the current study, it seems likely that this is also true for the Man family PTS transporters in the “Firm-5″ group. Notably, it was previously shown that 18 out of 27 tested carbohydrates were fermented only by a subset of the “Firm-5″ strains [30], so the specificity of these transporters merits further studies.

Phylotype-specific outer-surface protein families, which contain protein domains previously identified in secreted and cell surface proteins, also displayed extensive intra-phylotype diversity. Strain-specific variations in the number of genes and domains in each gene indicate that they evolve by duplication and divergence under diversifying selection. The RCC1-repeat domain proteins associated with the “Bifido” group showed particularly high levels of diversity.

Finally, we identified gene clusters associated with the biosynthesis of cell wall associated polysaccharides in both the “Firm-4″ and “Bifido” groups, where each strain encoded a unique gene set (except for the two closely related strains *B. indicum* and *B. coryneforme*). Cell wall associated polysaccharides have been shown to influence gut colonization and interactions with the immune response, suggesting that these genes may be of great importance for host-symbiont interactions in the honeybee gut [73]. Furthermore, exopolysaccharides are frequently involved in biofilm formation, which may provide resistance to the host immune response and exclude other bacteria from the habitat [74]. Thus, a particularly interesting question in this context is whether biofilms in the honeybee gut consist of members of different phylotypes, strains of the same phylotype or perhaps individual strains [75].

### Genetic exchange of mobile elements between members of the honeybee gut microbiota

Niche differentiation could also be the result of barriers to sequence exchange within or between phylotypes. We identified several large plasmids within the “Firm-5″ group, two of which were highly similar, indicative of a recent transfer. In contrast, a recent publication of two additional strains of the “Firm-5″ group did not identify any large plasmids, consistent with their dynamic nature [21]. Furthermore, the identification of prophages and CRISPR regions provides indirect evidence that bacteriophages are active in this environment. By including previously sequenced strains from honeybees and bumblebees, we found significant hits for 26 out of 781 spacer sequences. Interestingly, hits were mostly restricted to members of the same phylotype, suggesting that lactobacilli and bifidobacteria of honeybees consist of genetically well-separated phylotypes with distinct phage populations.

Within phylotypes, the emergence of resistance mechanisms to phage infections via the CRISPR-cas systems could prevent gene flow between certain strain combinations, and thereby further contribute to strain differentiation. Indeed, the phylogenies clearly supported the formation of micro-clusters within phylotypes, and only 2-9% of the phylotype core genes were significant for recombination. Furthermore, the ratio at which recombination versus mutation events contribute to sequence divergences in the core genes was in the range of 0.6 to 1.8, thus being low also for shorter recombination tracts. These values are similar to previous estimates for one of the lineages in *Lactobacillus sakeii* (*r/m* = 1.37) inferred to represent a clonal, specialized subpopulation [76].

### Parallels to the human gut microbiota

Several interesting parallels can be drawn between the honeybee microbiota and the human gut microbiome. Neither microbiome is vertically inherited and must therefore be established by colonization at each generation. The crop is sterile at eclosion, and the LAB microbiota starts to build up within minutes post-eclosion by trophallactic exchange with nestmates [10]. Similarly, the colonization of the infant gut begins during birth, where the mother’s vaginal and fecal microbiomes provide an important source of inoculum [77]. Interestingly, it has been shown that the human microbiome composition can change rapidly in response to a switch from a plant-based to an animal-based diet [78]. The ability to change the microbiota in response to herbivorous versus carnivorous diets is likely to have been a strongly selected trait in the evolution of humans, just like the ability to respond to changes in carbohydrate composition and concentrations in the nectar and pollen may have been of prime importance for the honeybee gut microbiota.

## Conclusions

The honeybee gut microbiota has been shown to be remarkably consistent, with a small number of phylotypes being repeatedly found to dominate the community. However, the current study revealed that the *Lactobacillus* and *Bifidobacterium* phylotypes consist of multiple strains with highly diverse gene content, indicating that the community is more complex than previously thought. Shared mobile elements and CRISPR spacer hits suggest that that members of the same phylotype exchange genetic material, which may provide possibilities for dynamic development of the phylotype accessory genomes. However, the low levels of homologous recombination suggest that such exchanges rarely affect the core gene contents of the strains, which will therefore continue to diverge.

We consider these results to be of specific interest for our understanding of the gut bacterial community of the honeybee and of general interest for our understanding of niche differentiation between bacteria adapted to the same habitat. The identification of unique outer surface structures, remarkably different repertoires of systems for the import of carbohydrate and resistance mechanisms to phage infections are some of the factors that may contribute to specialization, diversification and speciation. Experimental studies to elucidate whether the strains three phylotypes are spatially, temporally and/or functionally differentiated is an interesting avenue for future research.

## Methods

### Sample preparation and sequencing

Eleven strains of *Lactobacillus* and *Bifidobacterium* spp. were isolated from the crop of *Apis mellifera mellifera*, all collected from the same apiary during the summer season, in Jämtland, northern Sweden, as previously described [10,25,29]. The strains were cultured individually in MRS broth supplemented with 2% fructose and 0.1% L-cysteine. Extracted DNA was sequenced with 6 kb paired-end 454 and paired-end Illumina technologies. 454 sequencing was done on a 454 FLX Roche machine using Titanium chemistry and standard preparation for 6 kb libraries. Illumina sequencing was done on a Miseq instrument, using standard Illumina protocols for the preparation of paired-end libraries, generating 2x150 bp sequences from each fragment. All sequencing was done at MWG Eurofins Operon (Ebensburg, Germany).

### Genome assembly and annotation

The Illumina reads were trimmed using Trimmomatic [79]. Assemblies were done *de novo* with both 454 and Illumina data simultaneously with Newbler (454 Life Sciences Corp., Roche, Branford, CR 06405, US). The quality of the draft assemblies was evaluated using several strategies: the Illumina reads were mapped onto the draft genome sequences using bwa [80], and the coverage was calculated from the resulting bam-files using the depth command in samtools [81] and plotted using R [82]; GC content and skew was calculated and visualized with Artemis [83]; consistent versus inconsistent pair coverage was manually checked for all scaffolds using Consed [84]. Contigs smaller than 500 bp or with extremely low coverage were manually removed from the assemblies. The final contigs were concatenated prior to annotation, and the sequence was split and reverse-complemented as necessary to start with the *dnaA* gene as the first coding sequence.

An annotation pipeline was developed using the Diya framework [85], including the software Prodigal [86], tRNAscan [87] and RNAmmer [88]. GenePrimp was used to identify suspicious start/stop codons and pseudogenes [89]. Genes flagged by Geneprimp were manually inspected and edited using Artemis. Genes spanning contig borders were flagged as partial and excluded from further analyses.

In order to gain putative functional information from genes, which could not be functionally annotated using the pipeline, an hmmsearch as implemented in the perl-script pfam_scan.pl (ftp://ftp.sanger.ac.uk/pub/databases/Pfam/Tools) was used for domain prediction with the PFAM database [90]. Additionally, a COG classification [91] was run on all genes. For a gene to be assigned to a COG, an e-value below 0.01 to at least two proteins in the COG was required. Genes with significant hits in several were not assigned to any COG. For COGs affiliated with multiple categories, the first category was chosen.

Since PTS (Phosphotransferase systems) transporters were found to be numerous in several genomes, the annotation of these genes was manually refined. The complete proteome of each strain was blasted against the transporter classification database [55], and genes with hits to the Phosphotransfer-driven group translocators (family 4.A) with a relaxed e-value of 0.01 or lower were extracted. Among these, a positive PTS transporter annotation was inferred when the genes were found to be part of an operon, and with consistent PFAM predictions of PTS-domains. The annotated genes were further assigned to one of the 7 described PTS transporter families (4.A.1-7) based on their blast hits.

Putative genetic clusters involved in the biosynthesis of cell wall associated polysaccharides were inferred based on similarity to predicted *eps*-clusters in bifidobacteria [56] or genes within the plasmid-encoded *eps*-cluster from *Lactococcus lactis* [59], the presence of multiple glycosyl-transferases (based on pfam-domain predictions) and deviating GC content.

Plasmids were putatively identified based on read-pairs supporting circularization, gene content (e.g. plasmid replication genes) and read coverage. Contigs with uncertain status were analyzed as being part of the main chromosome. Prophage regions were inferred using ProphageFinder [92] with a conservative e-value of 0.001.

### Phylogenomics, gene content and recombination

In order to place the strains sequenced in the current study in a phylogenetic context with known species, completed genomes (Additional file 3: Table S2) were collected as follows. All complete genome sequences from strains classified within the Lactobacillaceae/Leuconostocaceae families or the genus *Bifidobacterium* were retrieved from Genbank. When several strains were available for a species, a single representative was chosen. For the Lactobacillaceae/Leuconostocaceae dataset, we included three outgroup strains: *Enterococcus faecalis*, *Lactococcus lactis* and *Streptococcus pyogenes*. Similarly, three outgroup strains were chosen for the *Bifidobacterium* dataset: *Mobiluncus curtisii, Arthrobacter phenanthrenivorans* and *Jonesia dentrificans,* together with *Gardnerella vaginalis*, since the taxonomic position of this species relative to the *Bifidobacterium* genus has previously been debated. Additionally, for the *Bifidobacterium* data set, 5 recently published genomes (three of which were draft genomes) of strains isolated from honeybees and bumblebees were included [31].

Orthologous genes were determined with OrthoMCL [93] for each of the two datasets, using the recommended inflation value (1.5). Clusters containing a single copy for each genome were used for the inference of core phylogenies. For the Lactobacillaceae/Leuconostocaceae data set, the 303 inferred single-copy orthologs were individually aligned at the protein level using mafft [94]. The alignments were pruned to remove gap sites present in 50% or more of the aligned sequences. A phylogeny was inferred from the concatenated alignment with RAxML using the PROTCATLG model [95]. One maximum likelihood tree was inferred with 100 bootstrap replicates. The same procedure was followed for the *Bifidobacterium* dataset, except that the aligned genes were back-translated to nucleotides prior to the phylogenetic inference, and RAxML was run using the GTRCAT model.

To evaluate the phylogenetic relationship between the strains in the current study and strains corresponding to the core gut microbiota of *Apis mellifera,* 16S rRNA gene sequences of the phylotypes “Firm-4″, “Firm-5″ and “Bifido” were selected from two studies [12,14] and downloaded from Genbank. The *uvrC* gene sequences corresponding to the “Firm” and “Bifido” phylotypes were extracted from [23]. Phylogenies were inferred with RAxML using the GTRCAT model. One slow best maximum likelihood tree was inferred with 100 rapid bootstrap replicates.

To search for evidence of homologous recombination in the core genome of both the Lactobacillaceae/Leuconostocaceae and *Bifidobacterium* datasets, individual gene phylogenies were inferred at the nucleotide level based on the back-translated protein alignment, using RAxML with the GTRCAT model. The trees were rooted with outgroups when possible using bioperl. Since the core phylogenies had indicated the presence of three strongly supported monophyletic groups (“Firm-4″, “Firm-5″, “Bifido”), the monophyly of each of these groups, as well as the “Bifido” subgroups (“Bifido-1″ and “Bifido-2″) was tested for the individual gene phylogenies using Newick utilities [96].

To estimate the core and accessory genomes of the “Firm-5″ and “Bifido” groups, clusters were extracted from the OrthoMCL predictions as follows. Group core clusters were defined as clusters containing a single gene copy for each of the strains in the group (and any number of additional genes from other strains). Group accessory clusters were defined as clusters containing a subset of strains from a group in any number of copies (and any number of additional genes from other strains). For the “Firm-4″ group, the core and accessory group clusters were not determined, since the group only had two members, which were not closely related. However, strain-specific accessory clusters were estimated as clusters containing one strain of the group, but not the other (and any number of genes from strains outside the group). To visualize the distribution of shared protein families between members of the same group, Venn diagrams were drawn with the R package “VennDiagram”.

To estimate the fraction of the group core genomes affected by recombination within the “Firm-5″ and “Bifido” groups, the genes of the group core clusters were aligned individually at the protein level with mafft. PhiPack [97] was run on each back-translated alignment, and recombination was inferred when a p-value below 0.01 was obtained with all three methods in the package (NSS, Maxchi and Phi).

To estimate the overall ratio at which recombination and mutation events (*r/m*) had generated substitutions, ClonalFrame [98] was run on genes previously used in multilocus sequence typing of *Lactobacillus casei* (*fusA, ileS, leuS, pyrG, recA* and *recG*) [99], and *Bifidobacterium* spp. (*fusA, ileS, gyrB, rplB* and *rpoB*). Clonalframe was run for 20000 generations and 100 generations between measures, and checked for convergence.

### CRISPR detection and analysis

Putative CRISPR regions were detected by using the tool CRISPRfinder [100]. The CRISPR-cas systems were classified by identification of the associated *cas* genes and their order following the classification proposed in [101].

CRISPR spacers were first compared through blast against the nr database and against a database containing only plasmid and phage sequences from the NCBI database. Second, the spacers of each CRISPR region were compared for hits against non-CRISPR regions in a database with masked spacer sequences, containing all the genomes analyzed in the current study and the recently published genomes of *Snodgrasella alvi* and *Gilliamella apicola* [20]. CRISPR region synteny was visualized with GenoPlotR [102], using tblastx (translated nucleotide blast) for the comparison files.

### Plots on genome synteny and specific genomic regions

All genome overview and gene comparison plots were constructed with GenoPlotR. Comparison files for the genome plots were generated with nucleotide blast, and filtered to exclude blast hits with a percentage identity below 80% or a hit length below 200 bp. For the “Firm-4″ strains, the length filter was set to 50 bp, based on the more distant relationship between these strains. Similarly, gene comparison plots of regions of specific interest were also plotted with GenoPlotR, but the comparison files were based on tblastx searches.

### Carbohydrate fermentation

In order to assess how the strains utilize carbohydrates present in nectars we performed sugar-fermentation patterns using the API 50 CHL system (bioMérieux, Lyon, France) in triplicates at 35 °C during 5 days of incubation.

### Data deposition

These Whole Genome Shotgun projects have been deposited at GenBank under the accession numbers: JXBX00000000, JWME00000000, JWMF00000000, JXJS00000000, JXBY00000000, JXLG00000000, JXLH00000000, JXLI00000000, JXJR00000000, JXBZ00000000, JXJQ00000000, where the versions described in this paper are JXBX01000000, JWME01000000, JWMF01000000, JXJS01000000, JXBY01000000, JXLG01000000, JXLH01000000, JXLI01000000, JXJR01000000, JXBZ01000000, JXJQ01000000. Additionally, the raw sequence data has been deposited at SRA, accessible via the BioProject numbers: PRJNA257132-PRJNA257134, PRJNA257136-139, PRJNA257141-142, PRJNA257182, PRJNA257185.

## Additional files

Additional file 1: Table S1. Sequencing data and assembly statistics. Additional file 2: Figure S1. GC skew and read coverage on final assembly. Example plots from strain Bin7. A) Circular genome plot showing GC content and GC skew. From inside out: 1. GC content, where content below average is shown in purple, and content above average is shown in yellow, 2. GC skew, 3. CDS on reverse strand, 4. CDS on leading strand. B) Coverage is indicated with a blue line, which was calculated with a moving average filter. Red vertical lines indicate the contig border positions. The small contigs with increased coverage at the end of the graph correspond to the rRNA gene operon. Additional file 3: Table S2. Accession numbers for reference genomes. Species names and accession numbers of genomes used for ortholog predictions with Ortho-MCL. Additional file 4: Figure S2. 16S rRNA and *uvrC* gene phylogenies of *Lactobacillus* strains from the NCFM clade. Gene phylogenies inferred from the A) 16S rRNA and B) *uvrC* gene sequences. The strains sequenced in the current study are highlighted in red. Bee gut microbiota sequences corresponding to phylotype “Firm4” are highlighted in green and phylotype “Firm-5” are highlighted in blue (taken from [12,14,23]). Additional sequences correspond to the members of the NCFM clade in the core phylogeny (see Figure 1). Only bootstrap support values above 70 are shown. Additional file 5: Figure S3. 16S rRNA and the *uvrC* phylogenies of *Bifidobacterium* strains. Gene phylogenies were inferred from the A) 16S rRNA and B) *uvrC* gene sequences. The strains sequenced in the current study are highlighted in red. Bee gut microbiota sequences corresponding to phylotype “Bifido” are shown in green (taken from [14,23]). Sequences from species isolated from the bumblebee are shown in dark blue, while species isolated from the honeybee are shown in light blue. Additional sequences were taken from the *Bifidobacterium* strains included in the core genome phylogeny (Figure 2). Only bootstrap support values above 70 are shown. Additional file 6: Figure S4. Venn diagram of shared protein clusters in the “Firm-5″ group. Numbers correspond to protein families of orthologous sequences, inferred with Ortho-MCL, plus singletons (proteins unique to a single strain). Additional file 7: Figure S5. Venn diagram of shared protein clusters in the “Bifido-2″ group. Numbers correspond to protein families of orthologous sequences, inferred with Ortho-MCL, plus singletons (proteins unique to a single strain). Additional file 8: Figure S6 Phylogeny of all two-domain RCC1-repeat domain proteins in the “Bifido” group. Strains from the "Bifido-1" subgroup are shown in yellow/red, strains from the "Bifido-2" subgroup are shown in blue, and *B. actinocoloniiforme* is indicated in green. Only bootstrap support values above 70 are shown. Additional file 9: Figure S7. Phylogeny of all three-domain RCC1-repeat domain proteins in the “Bifido” group. Strains from the "Bifido-1" subgroup are shown in yellow/red, strains from the "Bifido-2" subgroup are shown in blue. Only bootstrap support values above 70 are shown. Additional file 10: Figure S8. Distribution of PTS transporter families in “Firm-4″ and “Firm-5″ strains. The bars represent the number of genes assigned to each of the seven PTS transporter families currently described in the Transporter classification database [55]. Blue = *L. kullabergensis*, Green = *L. kimbladii*, Purple = *L. melliventris*, Orange = *L. helsingborgensis*, Yellow = *L. apis*, Brown = *L. mellifer*, Pink = *L. mellis*. Additional file 11: Figure S9. Phylogeny of the PTS Man transporter family proteins. Gene phylogenies were inferred from A) the IIC subunit and B) the IID subunit. Only bootstrap values above 70 are shown. Additional file 12: Table S3. Hits to the PFAM database for all genes annotated in putative *eps* regions. Additional file 13: Figure S10. Putative *eps* region in *B. actinocoloniiforme.* Comparison of gene content in the putative ortholog region of *B. indicum* and strain Bin7 is shown. Similarity was estimated with tblastx, using a length filter of 100 bp. Pink: dTDP rhamnose biosyntheis genes, Green: ABC transporter genes, Yellow: glycosyl-transferases or genes with orthology to known *eps* genes, Orange: glycosyl-hydrolases, Black: probable pseudogene, Grey: other genes with a putative function in polysaccharide biosynthesis. Light blue: putative catalase, dark blue: putative manganese transporter and repressor. For a complete list of protein domain predictions, see Additional file 12: Table S3. Additional file 14: Figure S11. Fermentation profiles. All carbohydrates found in nectar were evaluated (except stachyose). Black boxes indicate a positive fermentation, grey boxes indicate weak fermentation and white boxes indicate no fermentation. Additional file 15: Figure S12. Comparative analysis on CRISPR regions. A) the CRISPR regions in “Firm-5″ strains, B) the CRISPR regions in “Bifido” strains and *B. actinocoloniiforme*. Genes are shown as arrows, where *cas* genes are indicated in blue, pseudogenized *cas* genes are shown in green, genes involved in fatty acid biosynthesis are shown in yellow and other genes are shown in white. The similarity between genomes was inferred with tblastx and is shown with connecting grey lines, where darker lines indicate higher similarity. The topologies of the trees are as in Figures 1 and 2. Additional file 16: Table S4. CRISPR spacers and their targets. Spacers were extracted from all available genomes from the honeybee core gut microbiota (781 spacers), where the sub-set with significant hits is listed in the table (26 spacers).

## References

[1] Kearns CA, Inouye DW, Waser NM (1998). Endangered mutualisms: the concervation of plant-pollinator interactions. Annu Rev Ecol Syst.

[2] Klein AM, Vaissiere BE, Cane JH, Steffan-Dewenter I, Cunningham SA, Kremen C (2007). Importance of pollinators in changing landscapes for world crops. Proc Biol Sci.

[3] Evans JD, Schwarz RS (2011). Bees brought to their knees: microbes affecting honey bee health. Trends Microbiol.

[4] Cox-Foster DL, Conlan S, Holmes EC, Palacios G, Evans JD, Moran NA (2007). A metagenomic survey of microbes in honey bee colony collapse disorder. Science.

[5] Tian B, Fadhil NH, Powell JE, Kwong WK, Moran NA (2012). Long-term exposure to antibiotics has caused accumulation of resistance determinants in the gut microbiota of honeybees. mBio.

[6] Lee YK, Mazmanian SK (2010). Has the microbiota played a critical role in the evolution of the adaptive immune system?. Science.

[7] Round JL, Mazmanian SK (2009). The gut microbiota shapes intestinal immune responses during health and disease. Nat Rev Immunol.

[8] Walter J, Ley R (2011). The human gut microbiome: ecology and recent evolutionary changes. Annu Rev Microbiol.

[9] Koch H, Schmid-Hempel P (2011). Socially transmitted gut microbiota protect bumble bees against an intestinal parasite. Proc Natl Acad Sci U S A.

[10] Vasquez A, Forsgren E, Fries I, Paxton RJ, Flaberg E, Szekely L (2012). Symbionts as major modulators of insect health: lactic acid bacteria and honeybees. PLoS One.

[11] Forsgren E, Olofsson TC, Vasquez A, Fries I (2010). Novel lactic acid bacteria inhibiting Paenicillus larvae in honey bee larvae. Apidologie.

[12] Babendreier D, Joller D, Romeis J, Bigler F, Widmer F (2007). Bacterial community structures in honeybee intestines and their response to two insecticidal proteins. FEMS Microbiol Ecol.

[13] Jeyaprakash A, Hoy MA, Allsopp MH (2003). Bacterial diversity in worker adults of Apis mellifera capensis and Apis mellifera scutellata (Insecta: Hymenoptera) assessed using 16S rRNA sequences. J Invertebr Pathol.

[14] Martinson VG, Danforth BN, Minckley RL, Rueppell O, Tingek S, Moran NA (2011). A simple and distinctive microbiota associated with honey bees and bumble bees. Mol Ecol.

[15] Moran NA, Hansen AK, Powell JE, Sabree ZL (2012). Distinctive gut microbiota of honey bees assessed using deep sampling from individual worker bees. PLoS One.

[16] Sabree ZL, Hansen AK, Moran NA (2012). Independent studies using deep sequencing resolve the same set of core bacterial species dominating gut communities of honey bees. PLoS One.

[17] Ahn JH, Hong IP, Bok JI, Kim BY, Song J, Weon HY (2012). Pyrosequencing analysis of the bacterial communities in the guts of honey bees Apis cerana and Apis mellifera in Korea. J Microbiol.

[18] Konstantinidis KT, Tiedje JM (2007). Prokaryotic taxonomy and phylogeny in the genomic era: advancements and challenges ahead. Curr Opin Microbiol.

[19] Gevers D, Cohan FM, Lawrence JG, Spratt BG, Coenye T, Feil EJ (2005). Opinion: re-evaluating prokaryotic species. Nat Rev Microbiol.

[20] Engel P, Stepanauskas R, Moran NA (2014). Hidden diversity in honey bee gut symbionts detected by single-cell genomics. PLoS Genet.

[21] Kwong WK, Mancenido AL, Moran NA (2014). Genome sequences of lactobacillus sp. Strains wkB8 and wkB10, members of the Firm-5 Clade, from honey bee guts. Genome Announc.

[22] Nicolson SW, Thornburg RW (2007). Nectar Chemistry. Nectaries and Nectar.

[23] Engel P, Martinson VG, Moran NA (2012). Functional diversity within the simple gut microbiota of the honey bee. Proc Natl Acad Sci U S A.

[24] Edwards CG, Haag KM, Collins MD, Hutson RA, Huang YC (1998). Lactobacillus kunkeei sp. nov.: a spoilage organism associated with grape juice fermentations. J Appl Microbiol.

[25] Olofsson TC, Vasquez A (2008). Detection and identification of a novel lactic acid bacterial flora within the honey stomach of the honeybee Apis mellifera. Curr Microbiol.

[26] Anderson KE, Sheehan TH, Mott BM, Maes P, Snyder L, Schwan MR (2013). Microbial ecology of the hive and pollination landscape: bacterial associates from floral nectar, the alimentary tract and stored food of honey bees (Apis mellifera). PLoS One.

[27] Vasquez A, Olofsson TC, Sammataro D (2009). A scientific note on the lactic acid bacterial flora in honeybees in the USA - a comparison with bees from Sweden. Apidologie.

[28] Olofsson TC, Vasquez A, Sammataro D, Macharia J (2011). A scientific note on the lactic acid bacterial flora within the honeybee subspecies Apis mellifera (Buckfast), A.m. Scutellata, A.m. mellifera, and A.m. monticola. Apidologie.

[29] Butler E, Alsterfjord M, Olofsson TC, Karlsson C, Malmstrom J, Vasquez A (2013). Proteins of novel lactic acid bacteria from Apis mellifera mellifera: an insight into the production of known extra-cellular proteins during microbial stress. BMC Microbiol.

[30] Olofsson TC, Alsterfjord M, Nilson B, Butler E, Vasquez A (2014). Lactobacillus apinorum sp. nov., Lactobacillus mellifer sp. nov., Lactobacillus mellis sp. nov., Lactobacillus melliventris sp. nov., Lactobacillus kimbladii sp. nov., Lactobacillus helsingborgensis sp. nov. and Lactobacillus kullabergensis sp. nov., isolated from the honey stomach of the honeybee Apis mellifera. Int J Syst Evol Microbiol.

[31] Milani C, Lugli GA, Duranti S, Turroni F, Bottacini F, Mangifesta M (2014). Genomic encyclopedia of type strains of the genus bifidobacterium. Appl Environ Microbiol.

[32] Bottacini F, Milani C, Turroni F, Sanchez B, Foroni E, Duranti S (2012). Bifidobacterium asteroides PRL2011 genome analysis reveals clues for colonization of the insect gut. PLoS One.

[33] Lee JH, O’Sullivan DJ (2010). Genomic insights into bifidobacteria. Microbiol Mol Biol Rev.

[34] Kant R, Blom J, Palva A, Siezen RJ, de Vos WM (2011). Comparative genomics of Lactobacillus. Microb Biotechnol.

[35] Corby-Harris V, Maes P, Anderson KE (2014). The bacterial communities associated with honey bee (Apis mellifera) foragers. PLoS One.

[36] Gao B, Gupta RS (2012). Phylogenetic framework and molecular signatures for the main clades of the phylum Actinobacteria. Microbiol Mol Biol Rev.

[37] Scardovi V, Trovatelli LD (1969). New species of bifid bacteria from Apis mellifica L. and Apis indica F. A contribution to the taxonomy and biochemistry of the genus Bifidobacterium. Zentralbl Bakteriol Parasitenkd Infektionskr Hyg.

[38] Pedersen MB, Gaudu P, Lechardeur D, Petit MA, Gruss A (2012). Aerobic respiration metabolism in lactic acid bacteria and uses in biotechnology. Annu Rev Food Sci Technol.

[39] Elbein AD, Pan YT, Pastuszak I, Carroll D (2003). New insights on trehalose: a multifunctional molecule. Glycobiology.

[40] Goh YJ, Klaenhammer TR (2013). A functional glycogen biosynthesis pathway in Lactobacillus acidophilus: expression and analysis of the glg operon. Mol Microbiol.

[41] Schneewind O, Missiakas DM (2012). Protein secretion and surface display in Gram-positive bacteria. Philos Trans R Soc Lond B Biol Sci.

[42] Edelman SM, Lehti TA, Kainulainen V, Antikainen J, Kylvaja R, Baumann M (2012). Identification of a high-molecular-mass Lactobacillus epithelium adhesin (LEA) of Lactobacillus crispatus ST1 that binds to stratified squamous epithelium. Microbiology.

[43] Buck BL, Altermann E, Svingerud T, Klaenhammer TR (2005). Functional analysis of putative adhesion factors in Lactobacillus acidophilus NCFM. Appl Environ Microbiol.

[44] Walter J, Chagnaud P, Tannock GW, Loach DM, Dal Bello F, Jenkinson HF (2005). A high-molecular-mass surface protein (Lsp) and methionine sulfoxide reductase B (MsrB) contribute to the ecological performance of Lactobacillus reuteri in the murine gut. Appl Environ Microbiol.

[45] Roos S, Jonsson H (2002). A high-molecular-mass cell-surface protein from Lactobacillus reuteri 1063 adheres to mucus components. Microbiology.

[46] Hynonen U, Palva A (2013). Lactobacillus surface layer proteins: structure, function and applications. Appl Microbiol Biotechnol.

[47] Sun Z, Kong J, Hu S, Kong W, Lu W, Liu W (2013). Characterization of a S-layer protein from Lactobacillus crispatus K313 and the domains responsible for binding to cell wall and adherence to collagen. Appl Microbiol Biotechnol.

[48] Smit E, Oling F, Demel R, Martinez B, Pouwels PH (2001). The S-layer protein of Lactobacillus acidophilus ATCC 4356: identification and characterisation of domains responsible for S-protein assembly and cell wall binding. J Mol Biol.

[49] Antikainen J, Anton L, Sillanpaa J, Korhonen TK (2002). Domains in the S-layer protein CbsA of Lactobacillus crispatus involved in adherence to collagens, laminin and lipoteichoic acids and in self-assembly. Mol Microbiol.

[50] Hu S, Kong J, Sun Z, Han L, Kong W, Yang P (2011). Heterologous protein display on the cell surface of lactic acid bacteria mediated by the s-layer protein. Microb Cell Fact.

[51] Dramsi S, Magnet S, Davison S, Arthur M (2008). Covalent attachment of proteins to peptidoglycan. FEMS Microbiol Rev.

[52] Siezen R, Boekhorst J, Muscariello L, Molenaar D, Renckens B, Kleerebezem M (2006). Lactobacillus plantarum gene clusters encoding putative cell-surface protein complexes for carbohydrate utilization are conserved in specific gram-positive bacteria. BMC Genomics.

[53] Brinster S, Furlan S, Serror P (2007). C-terminal WxL domain mediates cell wall binding in Enterococcus faecalis and other gram-positive bacteria. J Bacteriol.

[54] Schachtsiek M, Hammes WP, Hertel C (2004). Characterization of Lactobacillus coryniformis DSM 20001 T surface protein Cpf mediating coaggregation with and aggregation among pathogens. Appl Environ Microbiol.

[55] Saier MH, Reddy VS, Tamang DG, Vastermark A (2014). The transporter classification database. Nucleic Acids Res.

[56] Hidalgo-Cantabrana C, Sanchez B, Milani C, Ventura M, Margolles A, Ruas-Madiedo P (2014). Genomic overview and biological functions of exopolysaccharide biosynthesis in Bifidobacterium spp. Appl Environ Microbiol.

[57] Boels IC, Beerthuyzen MM, Kosters MH, Van Kaauwen MP, Kleerebezem M, De Vos WM (2004). Identification and functional characterization of the Lactococcus lactis rfb operon, required for dTDP-rhamnose Biosynthesis. J Bacteriol.

[58] Bentley SD, Aanensen DM, Mavroidi A, Saunders D, Rabbinowitsch E, Collins M (2006). Genetic analysis of the capsular biosynthetic locus from all 90 pneumococcal serotypes. PLoS Genet.

[59] Van Kranenburg R, Marugg JD, Van S, Willem NJ, De Vos WM (1997). Molecular characterization of the plasmid-encoded eps gene cluster essential for exopolysaccharide biosynthesis in Lactococcus lactis. Mol Microbiol.

[60] Welman AD, Maddox IS (2003). Exopolysaccharides from lactic acid bacteria: perspectives and challenges. Trends Biotechnol.

[61] Sorek R, Lawrence CM, Wiedenheft B (2013). CRISPR-mediated adaptive immune systems in bacteria and archaea. Annu Rev Biochem.

[62] Moriya Y, Itoh M, Okuda S, Yoshizawa AC, Kanehisa M (2007). KAAS: an automatic genome annotation and pathway reconstruction server. Nucleic Acids Res.

[63] McFrederick QS, Cannone JJ, Gutell RR, Kellner K, Plowes RM, Mueller UG (2013). Specificity between lactobacilli and hymenopteran hosts is the exception rather than the rule. Appl Environ Microbiol.

[64] Frese SA, Benson AK, Tannock GW, Loach DM, Kim J, Zhang M (2011). The evolution of host specialization in the vertebrate gut symbiont Lactobacillus reuteri. PLoS Genet.

[65] Lee FJ, Rusch DB, Stewart FJ, Mattila HR, Newton IL (2015). Saccharide breakdown and fermentation by the honey bee gut microbiome. Environ Microbiol.

[66] Barker RJ (1977). Some carbohydrates found in pollen and pollen substitutes are toxic to honey bees. J Nutr.

[67] Sols A, Cadenas E, Alvarado F (1960). Enzymatic basis of mannose toxicity in honey bees. Science.

[68] Blatt J, Roces F (2001). Haemolymph sugar levels in foraging honeybees (Apis mellifera carnica): dependence on metabolic rate and in vivo measurement of maximal rates of trehalose synthesis. J Exp Biol.

[69] Barabote RD, Saier MH (2005). Comparative genomic analyses of the bacterial phosphotransferase system. Microbiol Mol Biol Rev.

[70] Zuniga M, Comas I, Linaje R, Monedero V, Yebra MJ, Esteban CD (2005). Horizontal gene transfer in the molecular evolution of mannose PTS transporters. Mol Biol Evol.

[71] O’Donnell MM, O’Toole PW, Ross RP (2013). Catabolic flexibility of mammalian-associated lactobacilli. Microb Cell Fact.

[72] Kotrba P, Inui M, Yukawa H (2001). Bacterial phosphotransferase system (PTS) in carbohydrate uptake and control of carbon metabolism. J Biosci Bioeng.

[73] Fanning S, Hall LJ, Cronin M, Zomer A, MacSharry J, Goulding D (2012). Bifidobacterial surface-exopolysaccharide facilitates commensal-host interaction through immune modulation and pathogen protection. Proc Natl Acad Sci U S A.

[74] Abee T, Kovacs AT, Kuipers OP, van der Veen S (2011). Biofilm formation and dispersal in Gram-positive bacteria. Curr Opin Biotechnol.

[75] Elias S, Banin E (2012). Multi-species biofilms: living with friendly neighbors. FEMS Microbiol Rev.

[76] Chaillou S, Lucquin I, Najjari A, Zagorec M, Champomier-Verges MC (2013). Population genetics of Lactobacillus sakei reveals three lineages with distinct evolutionary histories. PLoS One.

[77] Koenig JE, Spor A, Scalfone N, Fricker AD, Stombaugh J, Knight R (2011). Succession of microbial consortia in the developing infant gut microbiome. Proc Natl Acad Sci U S A.

[78] David LA, Maurice CF, Carmody RN, Gootenberg DB, Button JE, Wolfe BE (2014). Diet rapidly and reproducibly alters the human gut microbiome. Nature.

[79] Lohse M, Bolger AM, Nagel A, Fernie AR, Lunn JE, Stitt M (2012). RobiNA: a user-friendly, integrated software solution for RNA-Seq-based transcriptomics. Nucleic Acids Res.

[80] Li H, Durbin R (2009). Fast and accurate short read alignment with Burrows-Wheeler transform. Bioinformatics.

[81] Li H, Handsaker B, Wysoker A, Fennell T, Ruan J, Homer N (2009). Genome Project Data Processing S: the sequence alignment/map format and SAMtools. Bioinformatics.

[82] R Core Team (2013). R: a language and environment for statistical computing.

[83] Rutherford K, Parkhill J, Crook J, Horsnell T, Rice P, Rajandream MA (2000). Artemis: sequence visualization and annotation. Bioinformatics.

[84] Gordon D, Abajian C, Green P (1998). Consed: a graphical tool for sequence finishing. Genome Res.

[85] Stewart AC, Osborne B, Read TD (2009). DIYA: a bacterial annotation pipeline for any genomics lab. Bioinformatics.

[86] Hyatt D, Chen GL, Locascio PF, Land ML, Larimer FW, Hauser LJ (2010). Prodigal: prokaryotic gene recognition and translation initiation site identification. BMC Bioinformatics.

[87] Lowe TM, Eddy SR (1997). tRNAscan-SE: a program for improved detection of transfer RNA genes in genomic sequence. Nucleic Acids Res.

[88] Lagesen K, Hallin P, Rodland EA, Staerfeldt HH, Rognes T, Ussery DW (2007). RNAmmer: consistent and rapid annotation of ribosomal RNA genes. Nucleic Acids Res.

[89] Pati A, Ivanova NN, Mikhailova N, Ovchinnikova G, Hooper SD, Lykidis A (2010). GenePRIMP: a gene prediction improvement pipeline for prokaryotic genomes. Nat Methods.

[90] Bateman A, Birney E, Cerruti L, Durbin R, Etwiller L, Eddy SR (2002). The Pfam protein families database. Nucleic Acids Res.

[91] Tatusov RL, Galperin MY, Natale DA, Koonin EV (2000). The COG database: a tool for genome-scale analysis of protein functions and evolution. Nucleic Acids Res.

[92] Bose M, Barber RD (2006). Prophage Finder: a prophage loci prediction tool for prokaryotic genome sequences. In Silico Biol.

[93] Li L, Stoeckert CJ, Roos DS (2003). OrthoMCL: identification of ortholog groups for eukaryotic genomes. Genome Res.

[94] Katoh K, Misawa K, Kuma K, Miyata T (2002). MAFFT: a novel method for rapid multiple sequence alignment based on fast Fourier transform. Nucleic Acids Res.

[95] Stamatakis A (2006). RAxML-VI-HPC: maximum likelihood-based phylogenetic analyses with thousands of taxa and mixed models. Bioinformatics.

[96] Junier T, Zdobnov EM (2010). The Newick utilities: high-throughput phylogenetic tree processing in the UNIX shell. Bioinformatics.

[97] Bruen TC, Philippe H, Bryant D (2006). A simple and robust statistical test for detecting the presence of recombination. Genetics.

[98] Didelot X, Falush D (2007). Inference of bacterial microevolution using multilocus sequence data. Genetics.

[99] Diancourt L, Passet V, Chervaux C, Garault P, Smokvina T, Brisse S (2007). Multilocus sequence typing of Lactobacillus casei reveals a clonal population structure with low levels of homologous recombination. Appl Environ Microbiol.

[100] Grissa I, Vergnaud G, Pourcel C (2007). CRISPRFinder: a web tool to identify clustered regularly interspaced short palindromic repeats. Nucleic Acids Res.

[101] Makarova KS, Haft DH, Barrangou R, Brouns SJ, Charpentier E, Horvath P (2011). Evolution and classification of the CRISPR-Cas systems. Nat Rev Microbiol.

[102] Guy L, Kultima JR, Andersson SG (2010). genoPlotR: comparative gene and genome visualization in R. Bioinformatics.

