# Supplementary material for: Extensive intra-phylotype diversity in lactobacilli and bifidobacteria from the honeybee gut
Source: BMC Genomics. 2015 Apr 11;16(1):284. doi: 10.1186/s12864-015-1476-6 (PMC4449606; doi:10.1186/s12864-015-1476-6)
Supplement: Additional file 1: Table S1. — Sequencing data and assembly statistics. [file 12864_2015_1476_MOESM1_ESM.docx]

**Table S1: Assembly statistics**

| Strain | Size of largest scaffold^1^ (bp) | Total length of contigs (excl. plasmids) (bp) | Nb. contigs > 500bp | N50^2^ | Mean read coverage (per bp) |
| --- | --- | --- | --- | --- | --- |
| Bma6 | 1729379 | 1738065 | 14 | 943562 | 138x |
| Bin2 | 1003559 | 2087605 | 18 | 260533 | 101x |
| Bin7 | 2108801 | 2120336 | 11 | 1171278 | 94x |
| Hma3 | 2209299 | 2217062 | 14 | 571605 | 118x |
| *L. apis* | 1533540 | 1542091 | 15 | 282131 | 181x |
| *L. helsing-borgensis* | 1857416 | 1868619 | 25 | 209334 | 144x |
| *L. melliventris* | 1027378 | 1956081 | 18 | 248655 | 180x |
| *L. kimbladii* | 2116812 | 2130297 | 38 | 170214 | 138x |
| *L. kullabergensis* | 2070349 | 2079016 | 37 | 99399 | 178x |
| *L. mellis* | 1660656 | 1790038 | 17 | 443800 | 187x |
| *L. mellifer* | 1670295 | 1681465 | 18 | 221904 | 236x |

^1^ Excluding gaps

^2^ N50 specifies the length of the shortest contig that must be included to reach half the total assembly length, after sorting the contigs from longest to shortest and consecutively adding them together. Here, contigs annotated as plasmids were excluded.
