# Supplementary figures and images for: Extensive intra-phylotype diversity in lactobacilli and bifidobacteria from the honeybee gut
Source: BMC Genomics. 2015 Apr 11;16(1):284. doi: 10.1186/s12864-015-1476-6 (PMC4449606; doi:10.1186/s12864-015-1476-6)

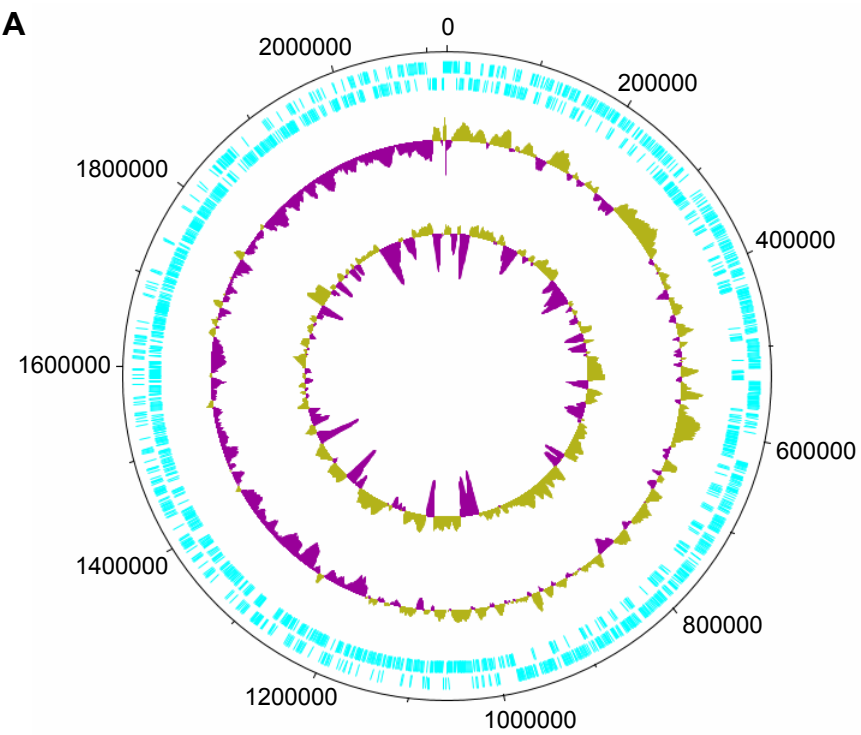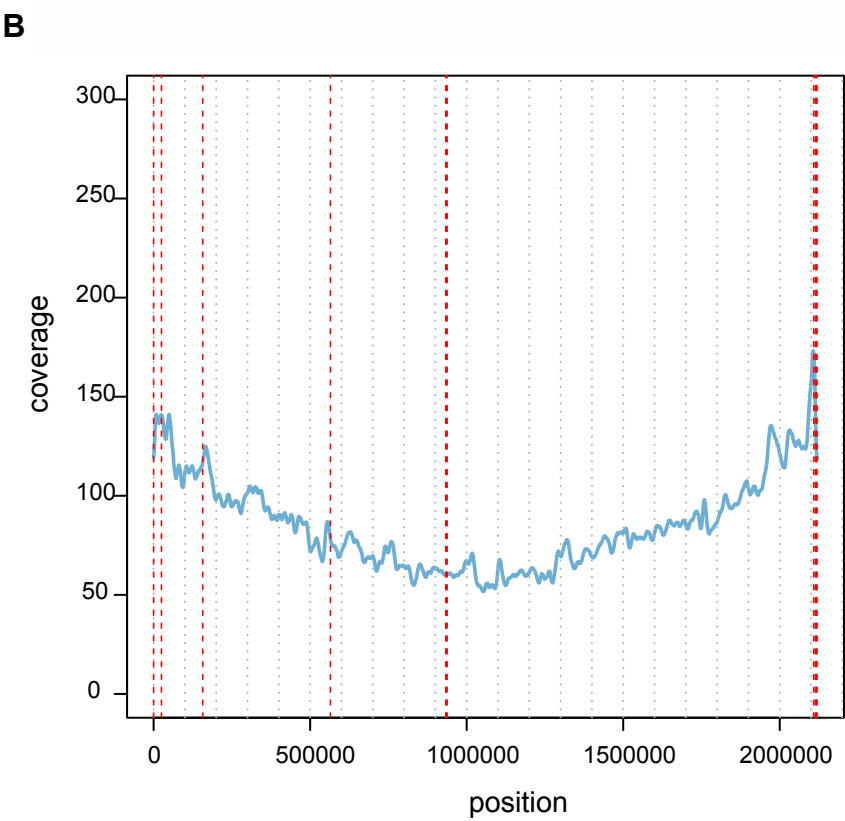

Supplement: Additional file 2: Figure S1. — GC skew and read coverage on final assembly. Example plots from strain Bin7. A) Circular genome plot showing GC content and GC skew. From inside out: 1. GC content, where content below average is shown in purple, and content above average is shown in yellow, 2. GC skew, 3. CDS on reverse strand, 4. CDS on leading strand. B) Coverage is indicated with a blue line, which was calculated with a moving average filter. Red vertical lines indicate the contig border positions. The small contigs with increased coverage at the end of the graph correspond to the rRNA gene operon. [file 12864_2015_1476_MOESM2_ESM.pdf]

**A**

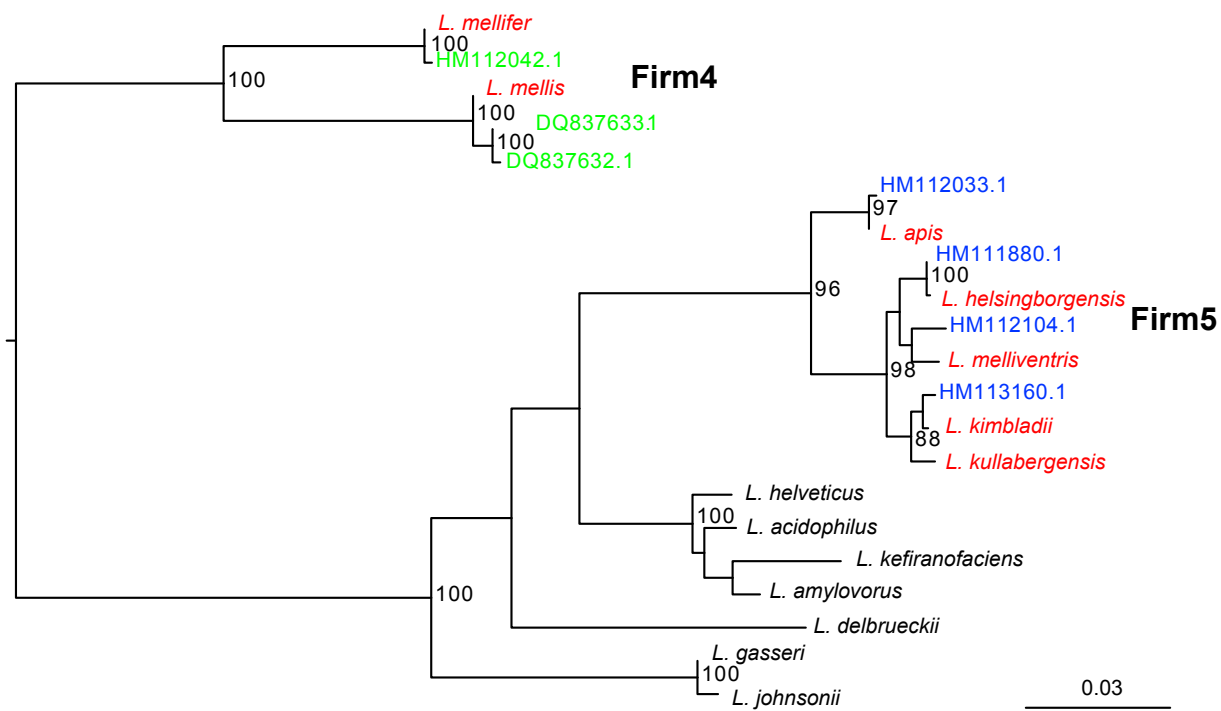

**B**

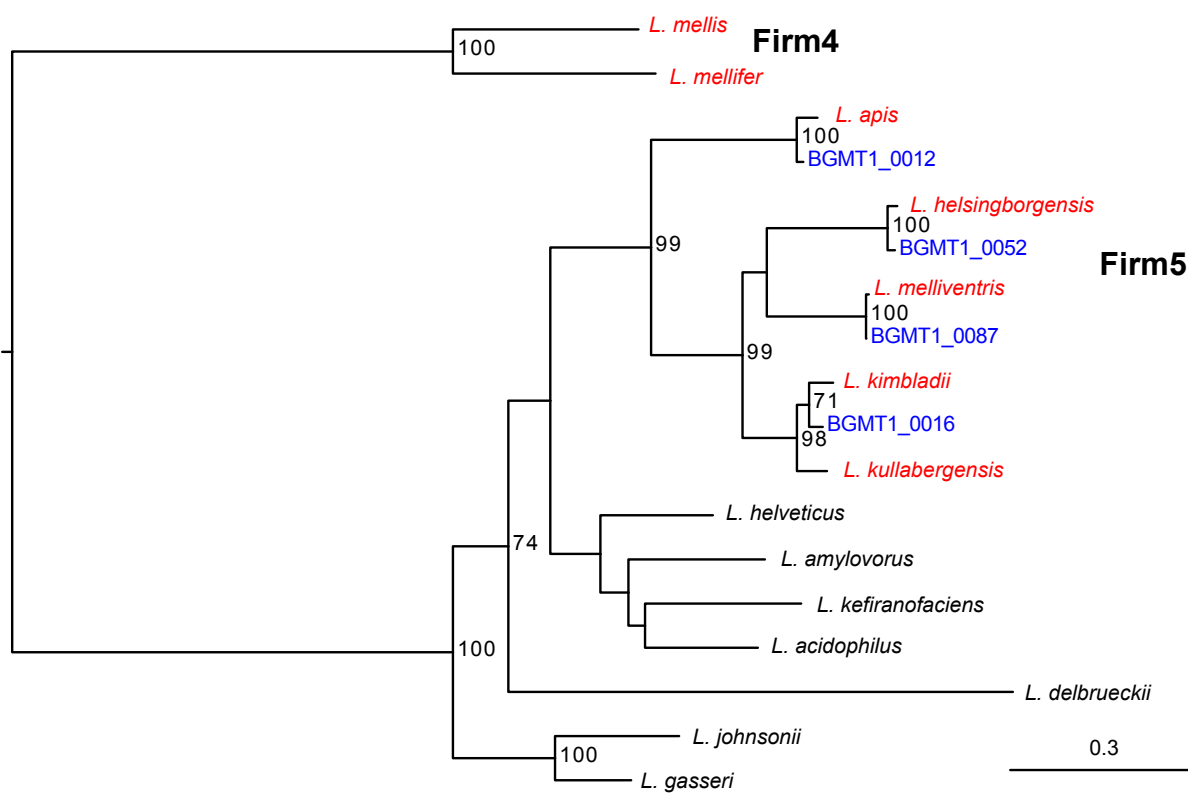

Supplement: Additional file 4: Figure S2. — 16S rRNA and uvrC gene phylogenies of Lactobacillus strains from the NCFM clade. Gene phylogenies inferred from the A) 16S rRNA and B) uvrC gene sequences. The strains sequenced in the current study are highlighted in red. Bee gut microbiota sequences corresponding to phylotype “Firm4” are highlighted in green and phylotype “Firm-5” are highlighted in blue (taken from [12,14,23]). Additional sequences correspond to the members of the NCFM clade in the core phylogeny (see Figure 1). Only bootstrap support values above 70 are shown. [file 12864_2015_1476_MOESM4_ESM.pdf]

A

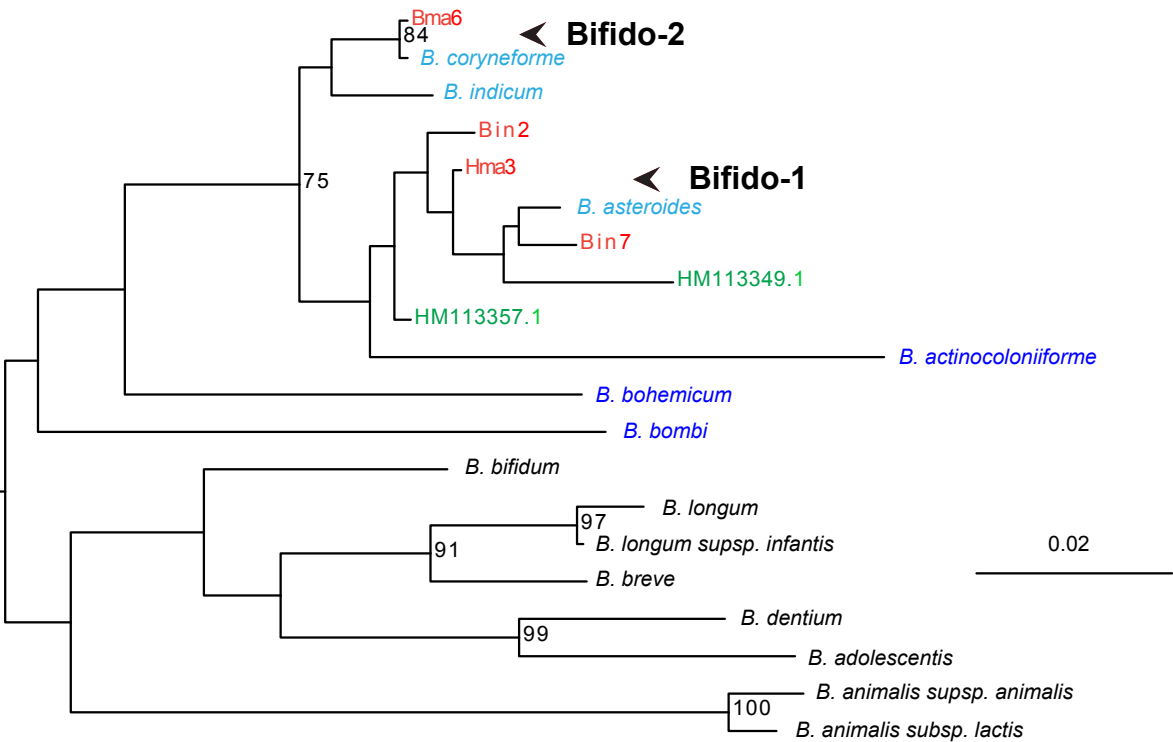

B

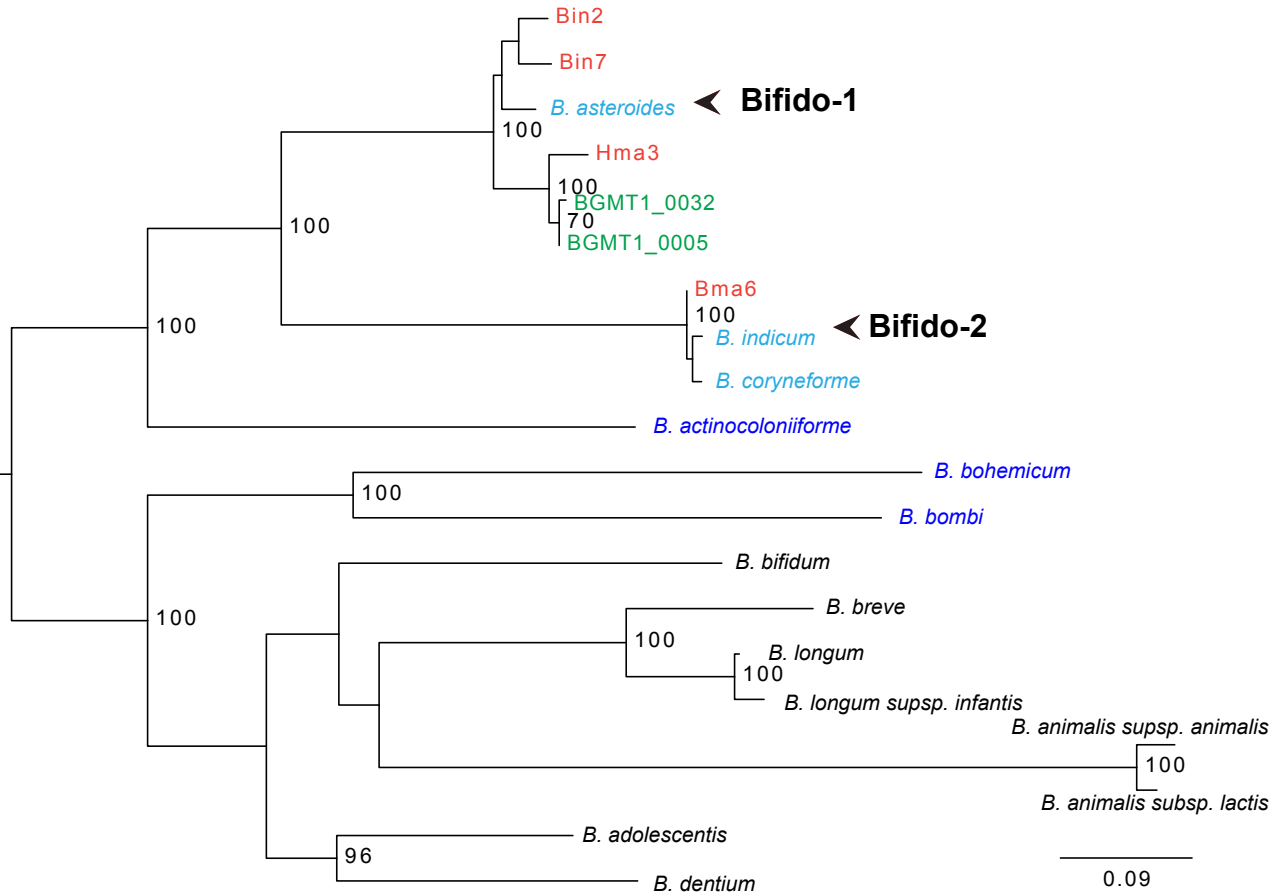

Supplement: Additional file 5: Figure S3. — 16S rRNA and the uvrC phylogenies of Bifidobacterium strains. Gene phylogenies were inferred from the A) 16S rRNA and B) uvrC gene sequences. The strains sequenced in the current study are highlighted in red. Bee gut microbiota sequences corresponding to phylotype “Bifido” are shown in green (taken from [14,23]). Sequences from species isolated from the bumblebee are shown in dark blue, while species isolated from the honeybee are shown in light blue. Additional sequences were taken from the Bifidobacterium strains included in the core genome phylogeny (Figure 2). Only bootstrap support values above 70 are shown. [file 12864_2015_1476_MOESM5_ESM.pdf]

*L. kullabergensis*

*L. melliventris*

*L. helsingborgensis*

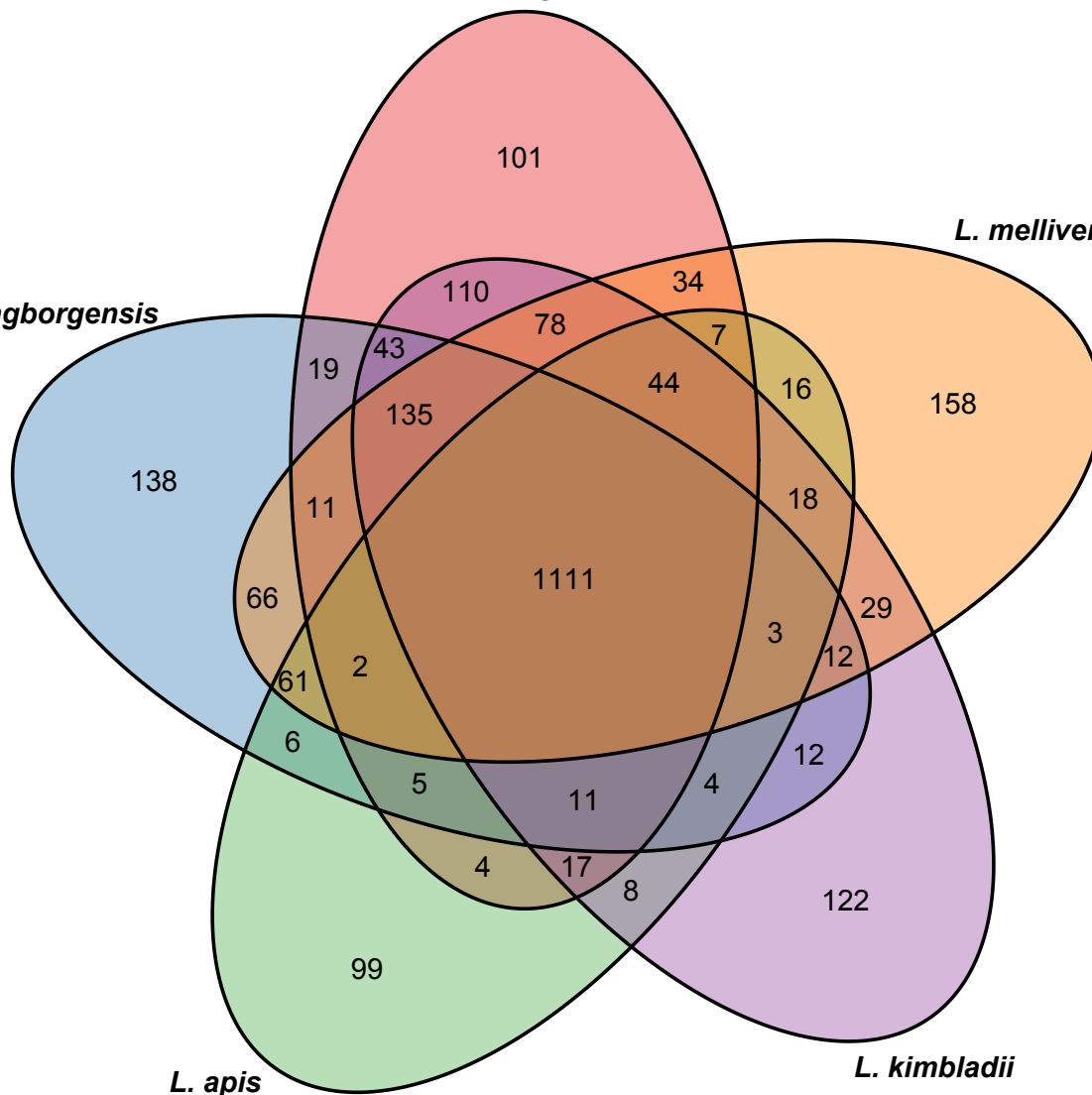

Supplement: Additional file 6: Figure S4. — Venn diagram of shared protein clusters in the “Firm-5″ group. Numbers correspond to protein families of orthologous sequences, inferred with Ortho-MCL, plus singletons (proteins unique to a single strain). [file 12864_2015_1476_MOESM6_ESM.pdf]

***B. coryneforme***

***B. indicum***

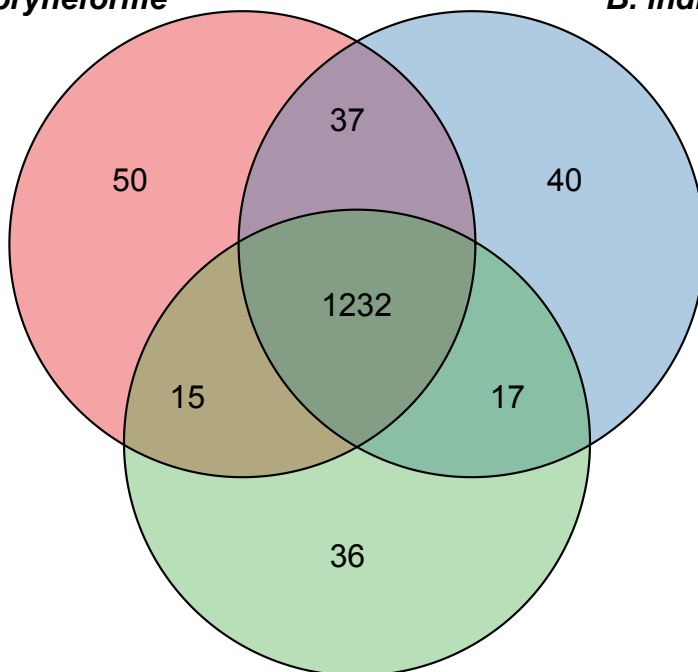

**Bma6**

Supplement: Additional file 7: Figure S5. — Venn diagram of shared protein clusters in the “Bifido-2″ group. Numbers correspond to protein families of orthologous sequences, inferred with Ortho-MCL, plus singletons (proteins unique to a single strain). [file 12864_2015_1476_MOESM7_ESM.pdf]

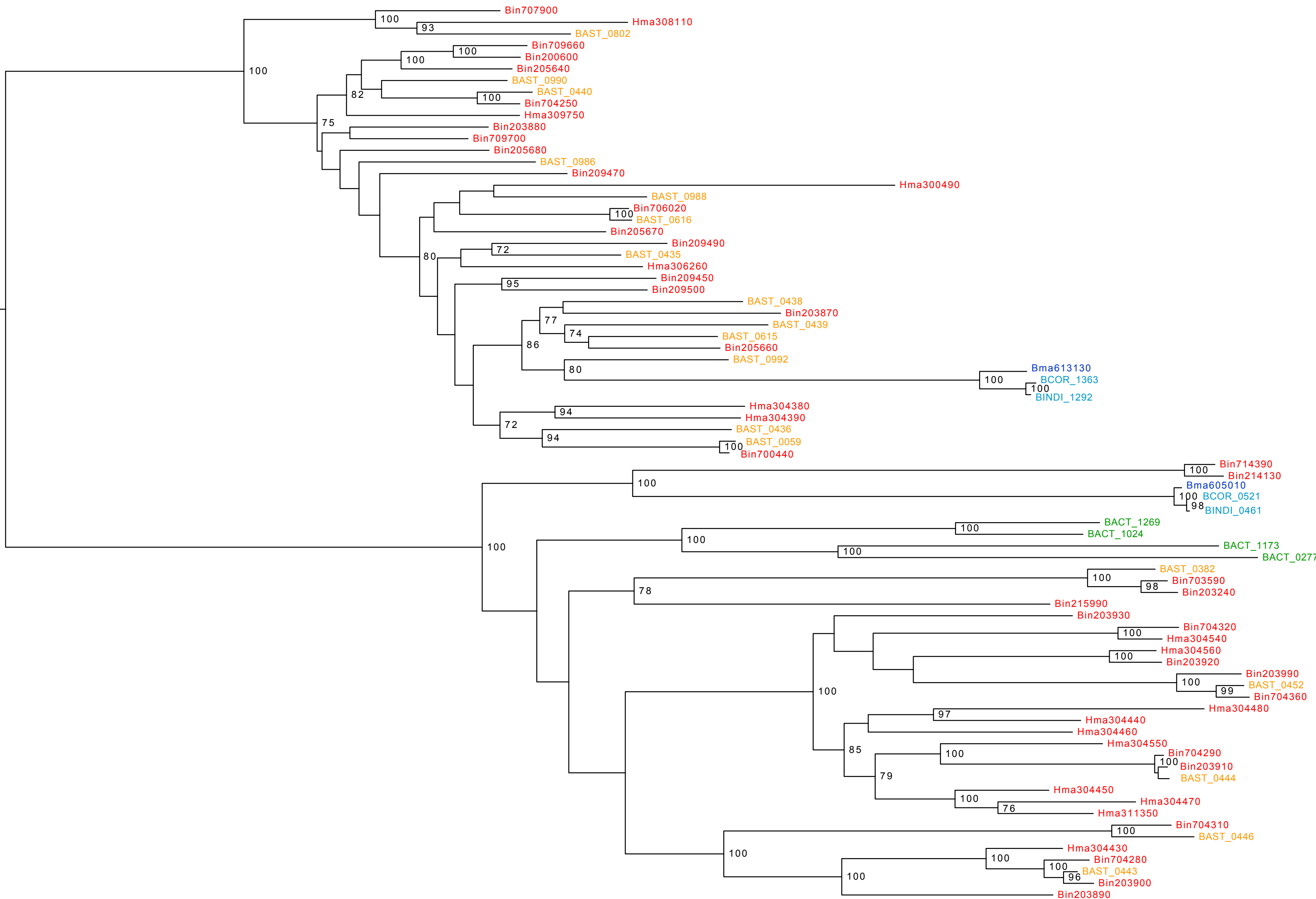

0.3

Supplement: Additional file 8: Figure S6 — Phylogeny of all two-domain RCC1-repeat domain proteins in the “Bifido” group. Strains from the "Bifido-1" subgroup are shown in yellow/red, strains from the "Bifido-2" subgroup are shown in blue, and B. actinocoloniiforme is indicated in green. Only bootstrap support values above 70 are shown. [file 12864_2015_1476_MOESM8_ESM.pdf]

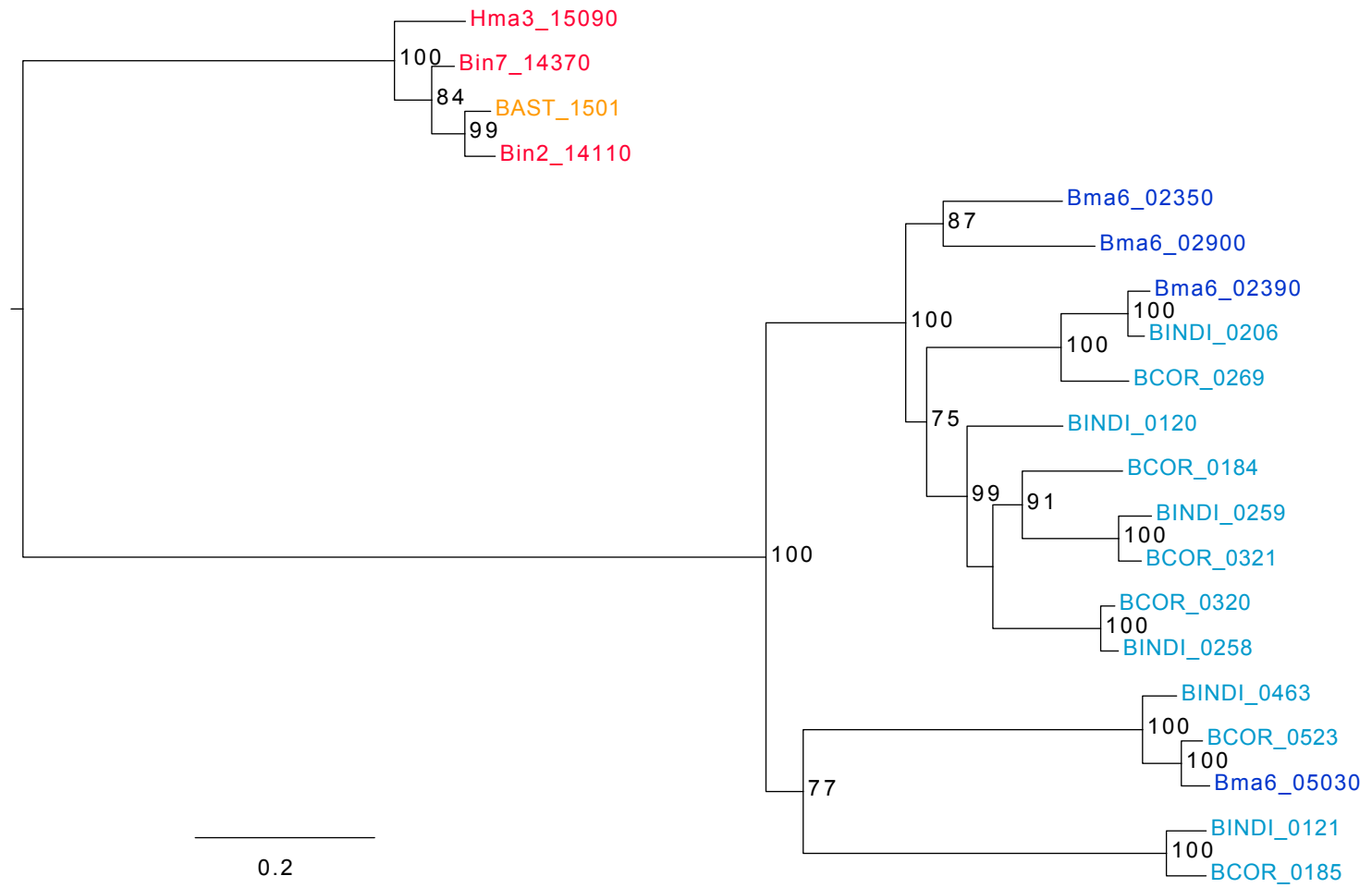

Supplement: Additional file 9: Figure S7. — Phylogeny of all three-domain RCC1-repeat domain proteins in the “Bifido” group. Strains from the "Bifido-1" subgroup are shown in yellow/red, strains from the "Bifido-2" subgroup are shown in blue. Only bootstrap support values above 70 are shown. [file 12864_2015_1476_MOESM9_ESM.pdf]

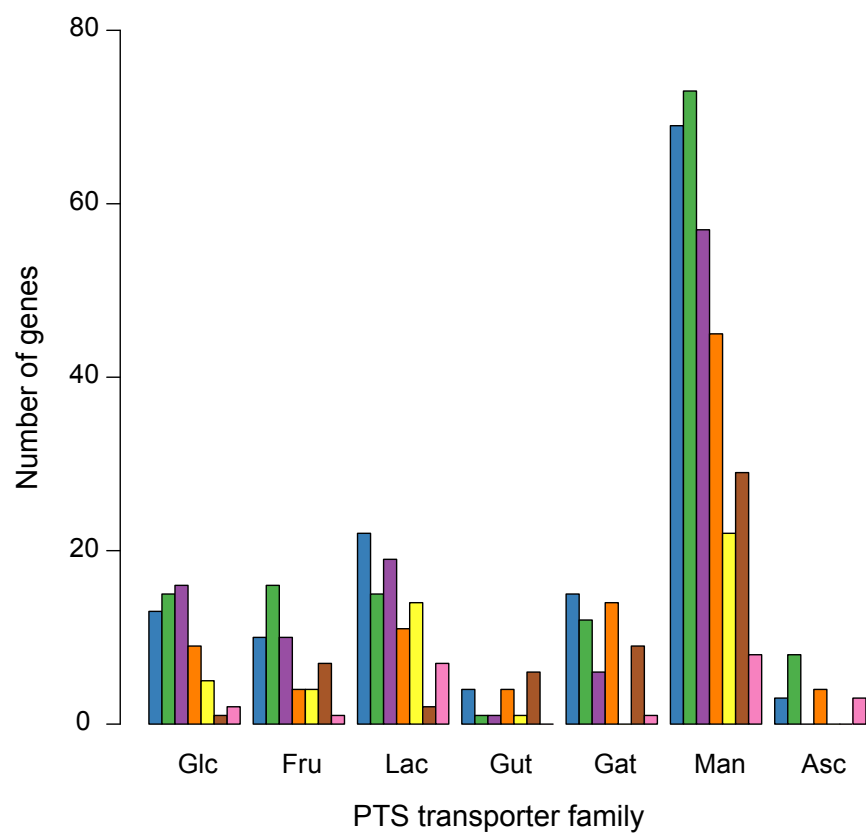

Supplement: Additional file 10: Figure S8. — Distribution of PTS transporter families in “Firm-4″ and “Firm-5″ strains. The bars represent the number of genes assigned to each of the seven PTS transporter families currently described in the Transporter classification database [55]. Blue = L. kullabergensis, Green = L. kimbladii, Purple = L. melliventris, Orange = L. helsingborgensis, Yellow = L. apis, Brown = L. mellifer, Pink = L. mellis. [file 12864_2015_1476_MOESM10_ESM.pdf]

A

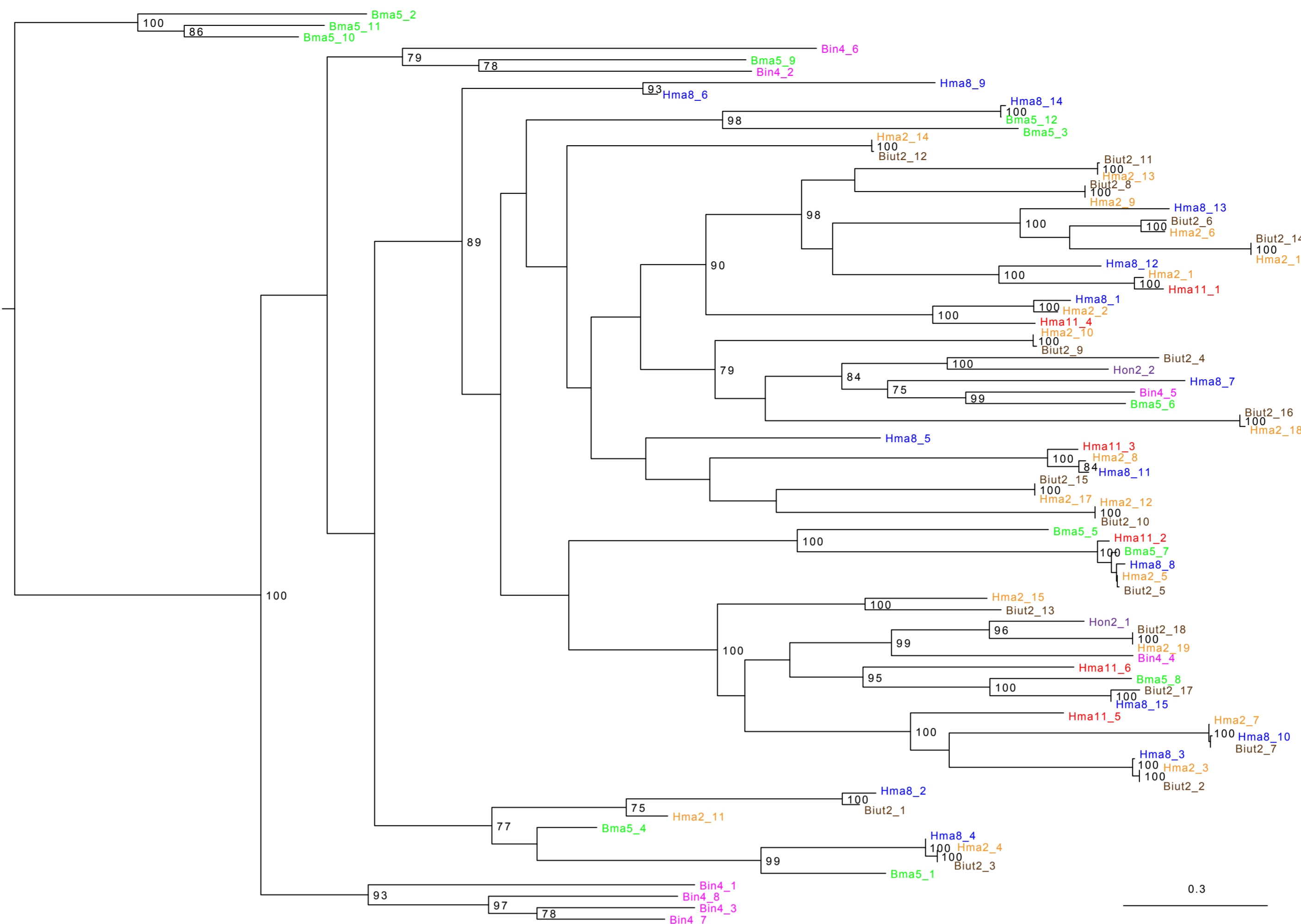

B

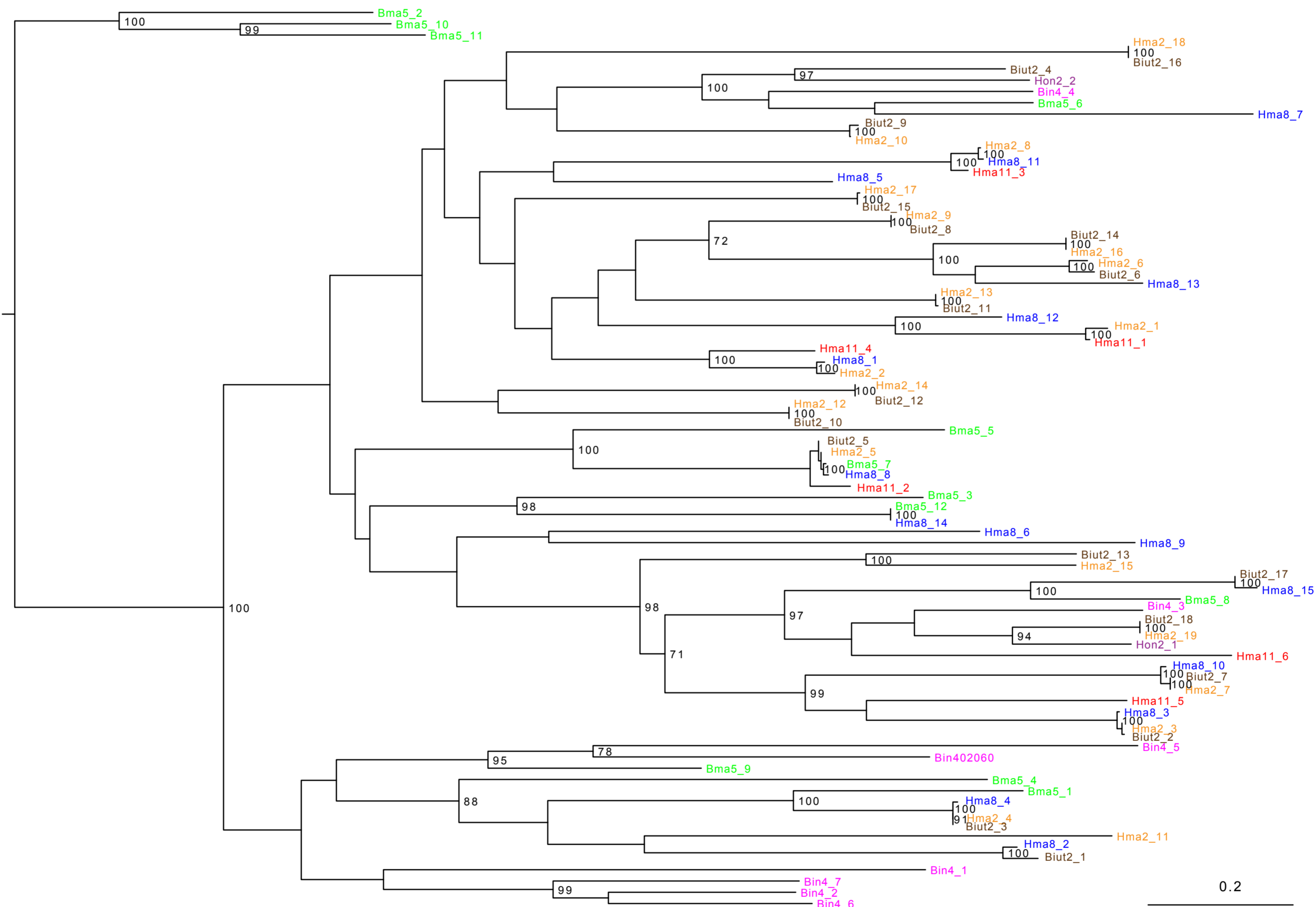

Supplement: Additional file 11: Figure S9. — Phylogeny of the PTS Man transporter family proteins. Gene phylogenies were inferred from A) the IIC subunit and B) the IID subunit. Only bootstrap values above 70 are shown. [file 12864_2015_1476_MOESM11_ESM.pdf]

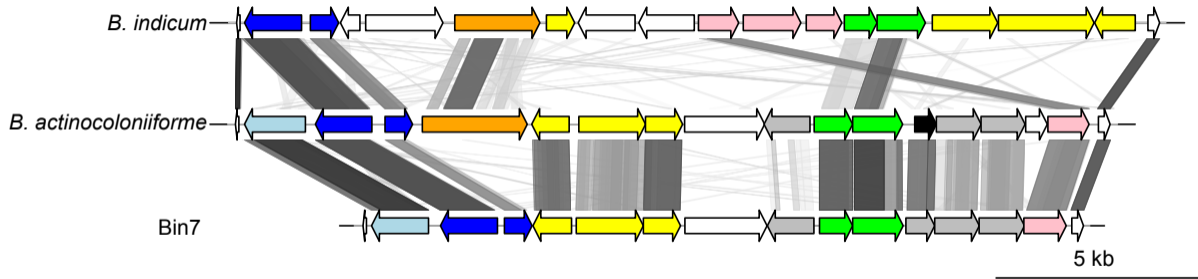

Supplement: Additional file 13: Figure S10. — Putative eps region in B. actinocoloniiforme. Comparison of gene content in the putative ortholog region of B. indicum and strain Bin7 is shown. Similarity was estimated with tblastx, using a length filter of 100 bp. Pink: dTDP rhamnose biosyntheis genes, Green: ABC transporter genes, Yellow: glycosyl-transferases or genes with orthology to known eps genes, Orange: glycosyl-hydrolases, Black: probable pseudogene, Grey: other genes with a putative function in polysaccharide biosynthesis. Light blue: putative catalase, dark blue: putative manganese transporter and repressor. For a complete list of protein domain predictions, see Additional file 12: Table S3. [file 12864_2015_1476_MOESM13_ESM.pdf]

**A**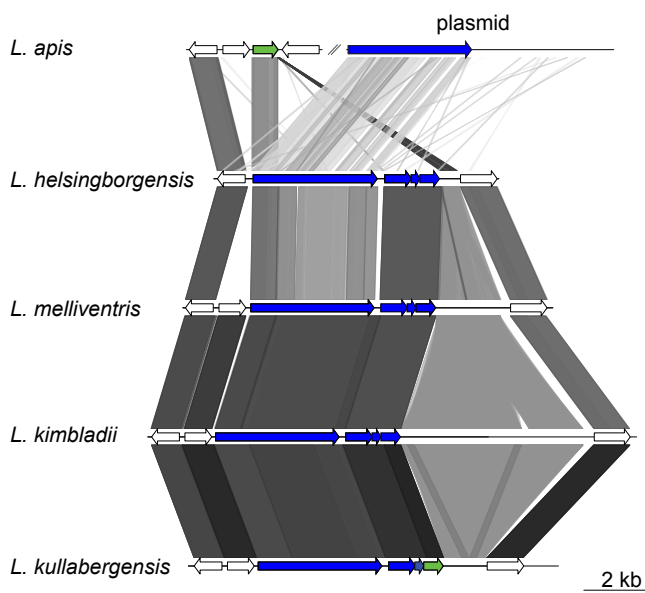**B**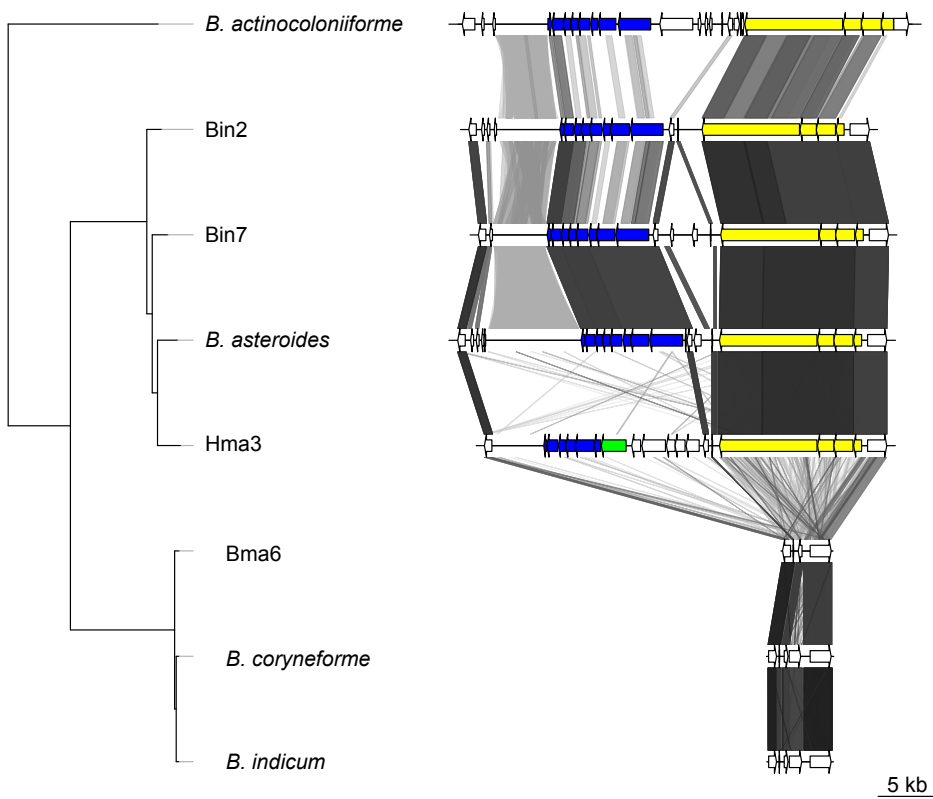

Supplement: Additional file 15: Figure S12. — Comparative analysis on CRISPR regions. A) the CRISPR regions in “Firm-5″ strains, B) the CRISPR regions in “Bifido” strains and B. actinocoloniiforme. Genes are shown as arrows, where cas genes are indicated in blue, pseudogenized cas genes are shown in green, genes involved in fatty acid biosynthesis are shown in yellow and other genes are shown in white. The similarity between genomes was inferred with tblastx and is shown with connecting grey lines, where darker lines indicate higher similarity. The topologies of the trees are as in Figures 1 and 2. [file 12864_2015_1476_MOESM15_ESM.pdf]
