# Supplementary material for: Extensive intra-phylotype diversity in lactobacilli and bifidobacteria from the honeybee gut
Source: BMC Genomics. 2015 Apr 11;16(1):284. doi: 10.1186/s12864-015-1476-6 (PMC4449606; doi:10.1186/s12864-015-1476-6)
Supplement: Additional file 3: Table S2. — Accession numbers for reference genomes. Species names and accession numbers of genomes used for ortholog predictions with Ortho-MCL. [file 12864_2015_1476_MOESM3_ESM.docx]

**Table S2: Accession numbers of genomes used for ortholog predictions with Ortho-MCL**

| Name | Accession number |
| --- | --- |
| *Lactobacillus acidophilus* | NC_021181 |
| *Lactobacillus amylovorus* | NC_014724 |
| *Lactobacillus brevis* | NC_008497 |
| *Lactobacillus buchneri* | NC_018610 |
| *Lactobacillus casei* | NC_008526 |
| *Lactobacillus delbrueckii* | NC_008054 |
| *Lactobacillus fermentum* | NC_010610 |
| *Lactobacillus gasseri* | NC_008530 |
| *Lactobacillus helveticus* | NC_010080 |
| *Lactobacillus johnsonii* | NC_017477 |
| *Lactobacillus kefiranofaciens* | NC_015602 |
| *Lactobacillus plantarum* | NC_021224 |
| *Lactobacillus reuteri* | NC_009513 |
| *Lactobacillus rhamnosus* | NC_017491 |
| *Lactobacillus sakei* | NC_007576 |
| *Lactobacillus salivarious* | NC_017481 |
| *Lactobacillus sanfranciscensis* | NC_015978 |
| *Leuconostoc carnosum* | NC_018673 |
| *Leuconostoc citreum* | NC_010471 |
| *Leuconostoc gasicomitatum* | NC_014319 |
| *Leuconostoc gelidum* | NC_018631 |
| *Leuconostoc kimchii* | NC_014136 |
| *Leuconostoc mesenteroides* | NC_008531 |
| *Oenococcus oeni* | NC_008528 |
| *Pediococcus claussenii* | NC_016605 |
| *Pediococcus pentosaceus* | NC_008525 |
| *Weissella koreensis* | NC_015759 |
| *Streptococcus_pyogenes* | NC_017053 |
| *Enterococcus faecalis* | NC_017316 |
| *Lactococcus lactis* | NC_009004 |
| *Bifidobacterium adolescentis* | NC_008618 |
| *Bidifobacterium animalis, subsp.lactis* | NC_012815 |
| *Bidifobacterium dentium* | NC_013714 |
| *Mobiluncus curtisii* | NC_014246 |
| *Arthrobacter phenanthrenivorans* | NC_015145 |
| *Bifidobacterium longum, subsp.infantis* | NC_017219 |
| *Bidifobacterium asteroides* | NC_018720 |
| *Bifidobacterium longum* | NC_010816 |
| *Jonesia denitrificans* | NC_013174 |
| *Gardnerella vaginalis* | NC_013721 |
| *Bifidobacterium bifidum* | NC_014616 |
| *Bifidobacterium breve* | NC_017218 |
| *Bidifobacterium animalis, subsp.animalis* | NC_017834 |
| *Bifidobacterium indicum* | CP006018 |
| *Bifidobacterium coryneforme* | CP007287 |
| *Bifidobacterium actinocoloniiforme* | JGYK00000000 |
| *Bifidobacterium bohemicum* | JGYP00000000 |
| *Bifidobacterium bombi* | ATLK00000000 |
