# Supplementary material for: Extensive intra-phylotype diversity in lactobacilli and bifidobacteria from the honeybee gut
Source: BMC Genomics. 2015 Apr 11;16(1):284. doi: 10.1186/s12864-015-1476-6 (PMC4449606; doi:10.1186/s12864-015-1476-6)
Supplement: Additional file 12: Table S3. — Hits to the PFAM database for all genes annotated in putative eps regions. [file 12864_2015_1476_MOESM12_ESM.docx]

**Table S3. PFAM domain predictions for proteins in putative *eps*-clusters**

| Locus-tag | Seq-length (aa) | PFAM identifier | Description | Hit alignment start | Hit alignment stop | e-value |
| --- | --- | --- | --- | --- | --- | --- |
| BAST_0260 | 224 | PF13472 | GDSL-like Lipase/Acylhydrolase family | 15 | 210 | 1.8E-14 |
| BAST_0261 | 461 | PF00083 | Sugar (and other) transporter | 20 | 459 | 2.7E-108 |
| BAST_0262 | 480 | PF00199 | Catalase | 6 | 386 | 4.9E-178 |
| - | 480 | PF06628 | Catalase-related immune-responsive | 408 | 473 | 1.4E-19 |
| BAST_0263 | 480 | PF01566 | Natural resistance-associated macrophage protein | 83 | 447 | 6.1E-124 |
| BAST_0264 | 233 | PF02742 | Iron dependent repressor, metal binding and dimerisation domain | 68 | 137 | 6.2E-32 |
| - | 233 | PF04023 | FeoA domain | 150 | 231 | 9.7E-7 |
| - | 233 | PF01325 | Iron dependent repressor, N-terminal DNA binding domain | 5 | 63 | 1.2E-14 |
| BAST_0265 | 585 | PF00534 | Glycosyl transferases group 1 | 409 | 524 | 6.4E-14 |
| BAST_0266 | 311 | PF00535 | Glycosyl transferase family 2 | 13 | 130 | 3.0E-25 |
| BAST_0267 | NO HIT in databases | |  |  |  |  |
| BAST_0268 | 386 | PF01757 | Acyltransferase family | 16 | 357 | 3.4E-21 |
| BAST_0269 | 301 | PF01061 | ABC-2 type transporter | 42 | 250 | 5.4E-21 |
| BAST_0270 | 424 | PF00005 | ABC transporter | 58 | 189 | 2.2E-20 |
| BAST_0271 | 356 | PF01370 | NAD dependent epimerase/dehydratase family | 26 | 264 | 2.6E-65 |
| BCOR_0215 | 480 | PF01566 | Natural resistance-associated macrophage protein | 83 | 447 | 2.9E-124 |
| BCOR_0216 | 238 | PF02742 | Iron dependent repressor, metal binding and dimerisation domain | 68 | 138 | 9.1E-30 |
| - | 238 | PF04023 | FeoA domain | 150 | 233 | 9.6E-10 |
| - | 238 | PF01325 | Iron dependent repressor, N-terminal DNA binding domain | 6 | 63 | 2.1E-15 |
| BCOR_0217 | NO HIT in databases | |  |  |  |  |
| BCOR_0218 | NO HIT in databases | |  |  |  |  |
| BCOR_0219 | 720 | PF01183 | Glycosyl hydrolases family 25 | 216 | 401 | 1.8E-19 |
| - | 720 | PF14200 | Ricin-type beta-trefoil lectin domain-like | 435 | 525 | 2.7E-21 |
| - | 720 | PF14200 | Ricin-type beta-trefoil lectin domain-like | 577 | 674 | 2.3E-14 |
| BCOR_0220 | 238 | PF00535 | Glycosyl transferase family 2 | 8 | 159 | 1.0E-16 |
| BCOR_0221 | 479 | PF09913 | Predicted membrane protein (DUF2142) | 28 | 432 | 5.4E-51 |
| BCOR_0222 | 467 | PF09913 | Predicted membrane protein (DUF2142) | 39 | 430 | 3.7E-43 |
| BCOR_0223 | 328 | PF01370 | NAD dependent epimerase/dehydratase family | 1 | 236 | 2.4E-66 |
| BCOR_0224 | 485 | PF04321 | RmlD substrate binding domain | 191 | 481 | 7.0E-70 |
| - | 485 | PF00908 | dTDP-4-dehydrorhamnose 3,5-epimerase | 12 | 183 | 5.4E-30 |
| BCOR_0225 | 299 | PF00483 | Nucleotidyl transferase | 3 | 238 | 1.6E-68 |
| BCOR_0226 | 275 | PF01061 | ABC-2 type transporter | 15 | 197 | 5.1E-20 |
| BCOR_0227 | 407 | PF00005 | ABC transporter | 55 | 186 | 8.7E-21 |
| - | 407 | PF14524 | Wzt C-terminal domain | 275 | 391 | 5.1E-13 |
| BCOR_0228 | 1363 | PF00535 | Glycosyl transferase family 2 | 831 | 992 | 3.8E-30 |
| - | 1363 | PF00535 | Glycosyl transferase family 2 | 1089 | 1220 | 5.1E-23 |
| - | 1363 | PF00535 | Glycosyl transferase family 2 | 180 | 330 | 2.2E-31 |
| BCOR_0229 | 342 | PF00535 | Glycosyl transferase family 2 | 9 | 127 | 5.9E-37 |
| Bin2_01950 | 480 | PF00199 | Catalase | 6 | 386 | 4.3E-178 |
| - | 480 | PF06628 | Catalase-related immune-responsive | 408 | 473 | 1.7E-19 |
| Bin2_01960 | 480 | PF01566 | Natural resistance-associated macrophage protein | 83 | 447 | 1.7E-124 |
| Bin2_01970 | 252 | PF04023 | FeoA domain | 151 | 249 | 2.1E-8 |
| - | 252 | PF01325 | Iron dependent repressor, N-terminal DNA binding domain | 5 | 63 | 1.9E-15 |
| - | 252 | PF02742 | Iron dependent repressor, metal binding and dimerisation domain | 68 | 137 | 3.9E-31 |
| Bin2_01980 | NO HIT in databases | |  |  |  |  |
| Bin2_01990 | 238 | PF00535 | Glycosyl transferase family 2 | 8 | 153 | 1.8E-19 |
| Bin2_02000 | 478 | PF09913 | Predicted membrane protein (DUF2142) | 36 | 431 | 5.0E-50 |
| Bin2_02010 | 467 | PF09913 | Predicted membrane protein (DUF2142) | 38 | 430 | 4.7E-44 |
| Bin2_02020 | 339 | PF01370 | NAD dependent epimerase/dehydratase family | 10 | 247 | 4.5E-67 |
| Bin2_02030 | 485 | PF00908 | dTDP-4-dehydrorhamnose 3,5-epimerase | 12 | 183 | 4.6E-30 |
| - | 485 | PF04321 | RmlD substrate binding domain | 191 | 482 | 5.8E-70 |
| Bin2_02040 | 299 | PF00483 | Nucleotidyl transferase | 3 | 238 | 5.0E-68 |
| Bin2_02050 | 275 | PF01061 | ABC-2 type transporter | 15 | 197 | 4.7E-20 |
| Bin2_02060 | 407 | PF14524 | Wzt C-terminal domain | 275 | 389 | 8.7E-14 |
| - | 407 | PF00005 | ABC transporter | 55 | 186 | 7.2E-21 |
| Bin2_02070 | 558 | PF13439 | Glycosyltransferase Family 4 | 12 | 172 | 7.4E-16 |
| Bin2_02080 | 1363 | PF00535 | Glycosyl transferase family 2 | 180 | 337 | 1.2E-30 |
| - | 1363 | PF00535 | Glycosyl transferase family 2 | 1089 | 1215 | 2.1E-21 |
| - | 1363 | PF00535 | Glycosyl transferase family 2 | 831 | 992 | 2.7E-30 |
| Bin2_02090 | 342 | PF00535 | Glycosyl transferase family 2 | 9 | 129 | 5.5E-36 |
| Bin7_02310 | 480 | PF00199 | Catalase | 6 | 386 | 1.6E-178 |
| - | 480 | PF06628 | Catalase-related immune-responsive | 408 | 473 | 2.6E-19 |
| Bin7_02320 | 480 | PF01566 | Natural resistance-associated macrophage protein | 83 | 447 | 9.6E-124 |
| Bin7_02330 | 233 | PF01325 | Iron dependent repressor, N-terminal DNA binding domain | 5 | 63 | 1.1E-14 |
| - | 233 | PF04023 | FeoA domain | 150 | 231 | 3.3E-8 |
| - | 233 | PF02742 | Iron dependent repressor, metal binding and dimerisation domain | 68 | 137 | 1.7E-31 |
| Bin7_02340 | 325 | PF00535 | Glycosyl transferase family 2 | 12 | 165 | 3.4E-18 |
| Bin7_02350 | 565 | PF00534 | Glycosyl transferases group 1 | 388 | 498 | 6.4E-13 |
| Bin7_02360 | 313 | PF00535 | Glycosyl transferase family 2 | 12 | 123 | 6.5E-25 |
| Bin7_02370 | NO HIT in databases | |  |  |  |  |
| Bin7_02380 | 392 | PF01757 | Acyltransferase family | 10 | 344 | 1.6E-20 |
| Bin7_02390 | 279 | PF01061 | ABC-2 type transporter | 20 | 228 | 3.9E-21 |
| Bin7_02400 | 425 | PF00005 | ABC transporter | 58 | 189 | 2.5E-20 |
| Bin7_02410 | 242 | PF01128 | 2-C-methyl-D-erythritol 4-phosphate cytidylyltransferase | 11 | 237 | 4.3E-37 |
| Bin7_02420 | 376 | PF01370 | NAD dependent epimerase/dehydratase family | 35 | 282 | 1.3E-32 |
| Bin7_02430 | 382 | PF04991 | LicD family | 124 | 215 | 7.9E-19 |
| Bin7_02440 | 357 | PF01370 | NAD dependent epimerase/dehydratase family | 27 | 265 | 6.8E-64 |
| BINDI_0149 | 480 | PF01566 | Natural resistance-associated macrophage protein | 83 | 447 | 2.9E-124 |
| BINDI_0150 | 238 | PF01325 | Iron dependent repressor, N-terminal DNA binding domain | 6 | 63 | 2.1E-15 |
| - | 238 | PF02742 | Iron dependent repressor, metal binding and dimerisation domain | 68 | 138 | 9.1E-30 |
| - | 238 | PF04023 | FeoA domain | 150 | 233 | 9.9E-10 |
| BINDI_0151 | NO HIT in databases | |  |  |  |  |
| BINDI_0152 | NO HIT in databases | |  |  |  |  |
| BINDI_0153 | 717 | PF01183 | Glycosyl hydrolases family 25 | 213 | 398 | 1.8E-19 |
| - | 717 | PF14200 | Ricin-type beta-trefoil lectin domain-like | 574 | 671 | 2.3E-14 |
| - | 717 | PF14200 | Ricin-type beta-trefoil lectin domain-like | 432 | 522 | 2.6E-21 |
| BINDI_0154 | 238 | PF00535 | Glycosyl transferase family 2 | 8 | 159 | 1.0E-16 |
| BINDI_0155 | 479 | PF09913 | Predicted membrane protein (DUF2142) | 28 | 432 | 5.4E-51 |
| BINDI_0156 | 467 | PF09913 | Predicted membrane protein (DUF2142) | 39 | 430 | 2.1E-43 |
| BINDI_0157 | 339 | PF01370 | NAD dependent epimerase/dehydratase family | 10 | 247 | 3.5E-68 |
| BINDI_0158 | 485 | PF04321 | RmlD substrate binding domain | 191 | 481 | 7.0E-70 |
| - | 485 | PF00908 | dTDP-4-dehydrorhamnose 3,5-epimerase | 12 | 183 | 5.4E-30 |
| BINDI_0159 | 299 | PF00483 | Nucleotidyl transferase | 3 | 238 | 1.6E-68 |
| BINDI_0160 | 275 | PF01061 | ABC-2 type transporter | 15 | 197 | 5.1E-20 |
| BINDI_0161 | 407 | PF00005 | ABC transporter | 55 | 186 | 8.7E-21 |
| - | 407 | PF14524 | Wzt C-terminal domain | 275 | 391 | 3.3E-13 |
| BINDI_0162 | 551 | PF00535 | Glycosyl transferase family 2 | 180 | 334 | 5.3E-32 |
| BINDI_0163 | 806 | PF00535 | Glycosyl transferase family 2 | 532 | 665 | 3.6E-23 |
| - | 806 | PF00535 | Glycosyl transferase family 2 | 274 | 435 | 2.3E-30 |
| BINDI_0164 | 342 | PF00535 | Glycosyl transferase family 2 | 9 | 127 | 3.3E-35 |
| Bma6_01810 | 480 | PF01566 | Natural resistance-associated macrophage protein | 83 | 447 | 6.6E-124 |
| Bma6_01820 | 238 | PF04023 | FeoA domain | 150 | 234 | 1.4E-9 |
| - | 238 | PF01325 | Iron dependent repressor, N-terminal DNA binding domain | 6 | 63 | 2.1E-15 |
| - | 238 | PF02742 | Iron dependent repressor, metal binding and dimerisation domain | 68 | 138 | 9.1E-30 |
| Bma6_01830 | NO HIT in databases | |  |  |  |  |
| Bma6_01840 | NO HIT in databases | |  |  |  |  |
| Bma6_01850 | 585 | PF01183 | Glycosyl hydrolases family 25 | 213 | 399 | 4.0E-23 |
| Bma6_01860 | 299 | PF00483 | Nucleotidyl transferase | 2 | 238 | 3.7E-70 |
| Bma6_01870 | 480 | PF00908 | dTDP-4-dehydrorhamnose 3,5-epimerase | 12 | 183 | 5.4E-29 |
| - | 480 | PF04321 | RmlD substrate binding domain | 191 | 476 | 1.0E-59 |
| Bma6_01880 | 338 | PF01370 | NAD dependent epimerase/dehydratase family | 9 | 246 | 5.2E-68 |
| Bma6_01890 | 254 | PF00005 | ABC transporter | 55 | 186 | 2.7E-20 |
| Bma6_01900 | 287 | PF01061 | ABC-2 type transporter | 29 | 236 | 9.8E-19 |
| Bma6_01910 | 280 | PF01370 | NAD dependent epimerase/dehydratase family | 5 | 224 | 4.9E-23 |
| Bma6_01920 | 352 | PF01757 | Acyltransferase family | 10 | 320 | 1.4E-19 |
| Bma6_01930 | 662 | PF05045 | Rhamnan synthesis protein F | 43 | 543 | 1.1E-130 |
| Bma6_01940 | 280 | PF00535 | Glycosyl transferase family 2 | 9 | 109 | 1.5E-15 |
| Bma6_01950 | 343 | PF00535 | Glycosyl transferase family 2 | 10 | 133 | 1.3E-36 |
| Hma3_02410 | 480 | PF06628 | Catalase-related immune-responsive | 408 | 473 | 9.7E-20 |
| - | 480 | PF00199 | Catalase | 6 | 386 | 2.5E-178 |
| Hma3_02420 | 480 | PF01566 | Natural resistance-associated macrophage protein | 83 | 447 | 2.2E-123 |
| Hma3_02430 | 242 | PF02742 | Iron dependent repressor, metal binding and dimerisation domain | 68 | 138 | 2.0E-31 |
| - | 242 | PF01325 | Iron dependent repressor, N-terminal DNA binding domain | 6 | 63 | 3.2E-15 |
| - | 242 | PF04023 | FeoA domain | 150 | 239 | 2.7E-8 |
| Hma3_02440 | 821 | PF01183 | Glycosyl hydrolases family 25 | 229 | 414 | 3.2E-20 |
| - | 821 | PF14200 | Ricin-type beta-trefoil lectin domain-like | 444 | 537 | 8.8E-17 |
| Hma3_02450 | 347 | PF00535 | Glycosyl transferase family 2 | 39 | 204 | 3.2E-23 |
| Hma3_02460 | 545 | PF14264 | Glucosyl transferase GtrII | 34 | 330 | 4.1E-13 |
| Hma3_02470 | 348 | PF00535 | Glycosyl transferase family 2 | 3 | 151 | 2.8E-18 |
| Hma3_02480 | NO HIT in databases | |  |  |  |  |
| Hma3_02490 | 381 | PF01757 | Acyltransferase family | 24 | 365 | 3.6E-28 |
| Hma3_02500 | NO HIT in databases | |  |  |  |  |
| Hma3_02510 | 696 | PF13516 | Leucine Rich repeat | 132 | 149 | 0.68 |
| - | 696 | PF13516 | Leucine Rich repeat | 113 | 129 | 1.5 |
| - | 696 | PF12799 | Leucine Rich repeats (2 copies) | 207 | 244 | 2.7E-11 |
| - | 696 | PF04122 | Putative cell wall binding repeat 2 | 475 | 573 | 6.4E-19 |
| - | 696 | PF04122 | Putative cell wall binding repeat 2 | 587 | 686 | 3.5E-17 |
| - | 696 | PF04122 | Putative cell wall binding repeat 2 | 369 | 459 | 2.0E-17 |
| Hma3_02520 | NO HIT in databases | |  |  |  |  |
| Hma3_02530 | 1006 | PF00535 | Glycosyl transferase family 2 | 5 | 144 | 9.1E-33 |
| - | 1006 | PF14393 | Domain of unknown function (DUF4422) | 387 | 631 | 9.2E-77 |
| - | 1006 | PF01501 | Glycosyl transferase family 8 | 667 | 928 | 1.1E-42 |
| Hma3_02540 | 394 | PF00984 | UDP-glucose/GDP-mannose dehydrogenase family, central domain | 198 | 289 | 6.6E-27 |
| - | 394 | PF03721 | UDP-glucose/GDP-mannose dehydrogenase family, NAD binding domain | 1 | 162 | 6.6E-36 |
| - | 394 | PF03720 | UDP-glucose/GDP-mannose dehydrogenase family, UDP binding domain | 308 | 381 | 2.2E-7 |
| Hma3_02550 | 626 | PF14393 | Domain of unknown function (DUF4422) | 13 | 254 | 4.5E-70 |
| - | 626 | PF01501 | Glycosyl transferase family 8 | 289 | 550 | 1.4E-47 |
| Hma3_02560 | 275 | PF01061 | ABC-2 type transporter | 19 | 214 | 1.1E-23 |
| Hma3_02570 | 419 | PF00005 | ABC transporter | 53 | 184 | 1.3E-19 |
| Hma3_02580 | 340 | PF01370 | NAD dependent epimerase/dehydratase family | 11 | 248 | 1.8E-63 |
| Bin4_11200 | 758 | PF09397 | Ftsk gamma domain | 686 | 749 | 9.4E-29 |
| - | 758 | PF01580 | FtsK/SpoIIIE family | 395 | 589 | 5.6E-59 |
| Bin4_11210 | 470 | PF14667 | Polysaccharide biosynthesis C-terminal domain | 324 | 459 | 3.4E-12 |
| - | 470 | PF01943 | Polysaccharide biosynthesis protein | 7 | 272 | 2.6E-46 |
| Bin4_11220 | NO HIT in databases | |  |  |  |  |
| Bin4_11230 | 320 | PF00535 | Glycosyl transferase family 2 | 6 | 151 | 1.5E-28 |
| Bin4_11240 | NO HIT in databases | |  |  |  |  |
| Bin4_11250 | 217 | PF14602 | Hexapeptide repeat of succinyl-transferase | 56 | 91 | 0.01 |
| - | 217 | PF14602 | Hexapeptide repeat of succinyl-transferase | 109 | 136 | 0.045 |
| - | 217 | PF00132 | Bacterial transferase hexapeptide (six repeats) | 163 | 197 | 1.0E-8 |
| Bin4_11260 | 383 | PF00534 | Glycosyl transferases group 1 | 189 | 357 | 3.1E-29 |
| - | 383 | PF13439 | Glycosyltransferase Family 4 | 13 | 181 | 3.1E-14 |
| Bin4_11270 | 392 | PF09314 | Domain of unknown function (DUF1972) | 2 | 190 | 1.2E-80 |
| - | 392 | PF00534 | Glycosyl transferases group 1 | 217 | 356 | 9.0E-5 |
| Bin4_11280 | 284 | PF04321 | RmlD substrate binding domain | 5 | 282 | 2.7E-97 |
| Bin4_11290 | 344 | PF01370 | NAD dependent epimerase/dehydratase family | 5 | 252 | 9.3E-65 |
| Bin4_11300 | 193 | PF00908 | dTDP-4-dehydrorhamnose 3,5-epimerase | 7 | 181 | 5.7E-65 |
| Bin4_11310 | 288 | PF00483 | Nucleotidyl transferase | 2 | 237 | 6.3E-71 |
| Bin4_11320 | 224 | PF02397 | Bacterial sugar transferase | 30 | 219 | 2.1E-66 |
| Bin4_11330 | 313 | PF01370 | NAD dependent epimerase/dehydratase family | 5 | 245 | 4.2E-57 |
| Bin4_11340 | 317 | PF03816 | Cell envelope-related transcriptional attenuator domain | 94 | 237 | 2.8E-46 |
| Bin4_11350 | NO HIT in databases | |  |  |  |  |
| Bin4_11360 | 237 | PF13614 | AAA domain | 52 | 198 | 6.0E-24 |
| Bin4_11370 | 258 | PF02706 | Chain length determinant protein | 4 | 141 | 1.2E-16 |
| - | 258 | PF13807 | G-rich domain on putative tyrosine kinase | 145 | 195 | 3.1E-10 |
| Bin4_11380 | 127 | PF06619 | Protein of unknown function (DUF1149) | 5 | 122 | 1.1E-23 |
| Bin4_11390 | 169 | PF00588 | SpoU rRNA Methylase family | 4 | 146 | 2.3E-32 |
| Bin4_11400 | 328 | PF00478 | IMP dehydrogenase / GMP reductase domain | 8 | 324 | 1.5E-95 |
| Hon211950 | 761 | PF09397 | Ftsk gamma domain | 688 | 752 | 2.5E-28 |
| - | 761 | PF01580 | FtsK/SpoIIIE family | 406 | 588 | 1.9E-58 |
| Hon211960 | 471 | PF01943 | Polysaccharide biosynthesis protein | 3 | 268 | 9.6E-47 |
| - | 471 | PF14667 | Polysaccharide biosynthesis C-terminal domain | 322 | 458 | 6.2E-24 |
| Hon211970 | 283 | PF04321 | RmlD substrate binding domain | 5 | 281 | 1.1E-97 |
| Hon211980 | 344 | PF01370 | NAD dependent epimerase/dehydratase family | 5 | 252 | 2.2E-63 |
| Hon211990 | 193 | PF00908 | dTDP-4-dehydrorhamnose 3,5-epimerase | 7 | 181 | 1.2E-67 |
| Hon212000 | 298 | PF00483 | Nucleotidyl transferase | 2 | 238 | 2.5E-71 |
| Hon212010 | NO HIT in databases | |  |  |  |  |
| Hon212020 | 311 | PF00535 | Glycosyl transferase family 2 | 10 | 171 | 2.7E-20 |
| Hon212030 | 283 | PF00535 | Glycosyl transferase family 2 | 7 | 163 | 2.9E-9 |
| Hon212040 | 231 | PF02397 | Bacterial sugar transferase | 37 | 228 | 3.2E-66 |
| Hon212050 | 134 | PF06619 | Protein of unknown function (DUF1149) | 1 | 121 | 5.0E-26 |
| Hon212060 | 169 | PF00588 | SpoU rRNA Methylase family | 4 | 146 | 2.1E-30 |
| Hon212070 | 341 | PF00478 | IMP dehydrogenase / GMP reductase domain | 8 | 325 | 1.0E-96 |
