# Supplementary material for: Extensive intra-phylotype diversity in lactobacilli and bifidobacteria from the honeybee gut
Source: BMC Genomics. 2015 Apr 11;16(1):284. doi: 10.1186/s12864-015-1476-6 (PMC4449606; doi:10.1186/s12864-015-1476-6)
Supplement: Additional file 16: Table S4. — CRISPR spacers and their targets. Spacers were extracted from all available genomes from the honeybee core gut microbiota (781 spacers), where the sub-set with significant hits is listed in the table (26 spacers). [file 12864_2015_1476_MOESM16_ESM.docx]

**Table S4. CRISPR-spacers and their targets**

| Spacer origin phylotype group/origin^1^ | Spacer origin strain | Spacer^2^ | Spacer target group/species^1^ | Spacer target strain | Spacer target gene | Hit annotation | Fraction identical bases |
| --- | --- | --- | --- | --- | --- | --- | --- |
| “Firm-5” | *L.kullabergensis* | 15 | “Firm-5” | *L.kimbladii* | Hma2_07630 | Putative phage integrase | 30/30 |
|  |  | 18 | “Firm-5” | *L.kullabergensis* | Biut2_07630 | Putative tail protein | 29/30 |
|  | *L.kimbladii* | 42 | “Firm-5” | *L.kullabergensis* | Biut2_07460 | Phage protein F-like protein | 28/30 |
| “Bifido-1” | Bin2 | 10 | “Bifido-1” | *B. asteroides* | BAST_1036, BAST_1037 | Between “hypothetical” and “phage protein” | 32/34 |
|  |  | 81 | “Bifido-1” | Bin7 | Bin7_08940 | Hypothetical protein | 31/33 |
|  | Bin7 | 20 | “Bifido-1” | Bin7 | Bin7_14390 | RCC1-domain protein | 31/33 |
|  | Hma3 | 3 | “Bumble bee” | *B. actino-coloniiforme* | BACT_1060 | glycoside hydrolase, family 25 | 31/33 |
|  |  | 21 | “Bifido-2” | *B. coryneforme* | BCOR_0871, BCOR_0872 | Hypothetical protein, hypothetical protein | 34/34 |
|  |  | 39 | “Bifido-2” | *B. indicum* | BINDI_0915 | Type II cytosine-5 DNA methyltransferase | 31/34 |
|  |  | 40 | “Bumble bee” | *B. bombi* | BBOMB_1044,BBOMB_1045 | HNH endonuclease domain-containing protein, hypothetical protein | 31/34 |
|  | *B. asteroides* | 5 | “Bifido-1” | Bin7 | Bin7_08940 | Hypothetical protein | 32/34 |
|  |  | 12 | "Bifido-1" | Bin7 | Bin7_08940 | Hypothetical protein | 33/33 |
|  |  | 45 | “Bifido-2” | *B. coryneforme* | BCOR_0870, BCOR_0871 | Hypothetical protein, Hypothetical protein | 29/34 |
|  |  | 126 | “Bumble bee” | *B. bombi* | BBOMB_1031 | phage-related minor tail protein | 27/33 |
| Gilliamella | wkB11 | NA^3^ | Gilliamella | wkB30 | GAPWKB30_0344 | single-strand DNA-binding protein | 32/32 |
|  |  | NA^3^ | Gilliamella | wkB30 | GAPWKB30_0333 | hypothetical protein | 32/32 |
|  |  | NA^3^ | Gilliamella | wkB11 | GAPWKB11_0813, GAPWKB11_0814 | hypothetical protein, hypothetical protein | 29/32 |
|  |  | NA^3^ | Gilliamella | wkB11 | GAPWKB11_0761, GAPWKB11_0762 | EF hand domain protein, Pyridoxine biosynthesis glutamine amidotransferase, glutaminase subunit | 32/32 |
|  |  | NA^3^ | Gillimella | wkB30 | GAPWKB30_0341 | hypothetical protein | 32/32 |
|  | wkB1 | 9^4^ | “Firm-5” | *L. apis* (plasmid) | Hma11_14960 | hypothetical protein | 25/32 |
| “Bumble bee” | *B. actinocoloniiforme* | 1 | “Bumble bee” | *B. actinocoloniiforme* | BACT_1278 | L-arabinose isomerase | 32/32 |
|  |  | 26 | “Bumble bee” | *B. actinocoloniiforme* | BACT_1066 | putative phage tail protein, probable tape-measure protein | 30/32 |
|  |  | 53 | “Bumble bee” | *B. actinocoloniiforme* | BACT_1064 | hypothetical protein | 30/32 |
|  | *B. bombi* | 5 | “Bumble bee” | *B. actinocoloniiforme* | BACT_0494 | phage protein Gp19/Gp15/Gp42 | 28/28 |
|  | *B.bohemicum* | NA^3^ | NA | *B. longum* | BLIJ_0882,BLIJ_0883 | hypothetical protein, D-tyrosyl-tRNA deacylase | 30/34 |
|  |  | NA |  | *B. longum*^5^ | BLIJ_0882/BLD_0184 | hypothetical protein/transcriptional regulator | 34/35 |

^1^ Phylotype group affilition according to previous publications given when appropriate. "Bifido-1" and "Bifido-2" refer to the groups shown in Fig.2, “Bumble bee” refers to all *Bifidobacterium* strains isolated from the bumblebee

^2^ The position of the spacer in the repeat-spacer array is given, in the cases where there is only a single CRISPR region in the genome

^3^ wkB11 and *B.bohemicum* are draft genome sequences, with multiple assembly contigs and CRISPR regions according to CRISPR-finder.

^4^ wkB1 contains 8 CRISPR regions according to CRISPR-finder, but only one region encodes cas-genes upstream; The spacer hit is from this region

^5^ Equally good hits to both *B.longum* strains included in the current study
