# Supplementary material for: Extensive intra-phylotype diversity in lactobacilli and bifidobacteria from the honeybee gut
Source: BMC Genomics. 2015 Apr 11;16(1):284. doi: 10.1186/s12864-015-1476-6 (PMC4449606; doi:10.1186/s12864-015-1476-6)
Supplement: Additional file 14: Figure S11. — Fermentation profiles. All carbohydrates found in nectar were evaluated (except stachyose). Black boxes indicate a positive fermentation, grey boxes indicate weak fermentation and white boxes indicate no fermentation. [file 12864_2015_1476_MOESM14_ESM.pdf]

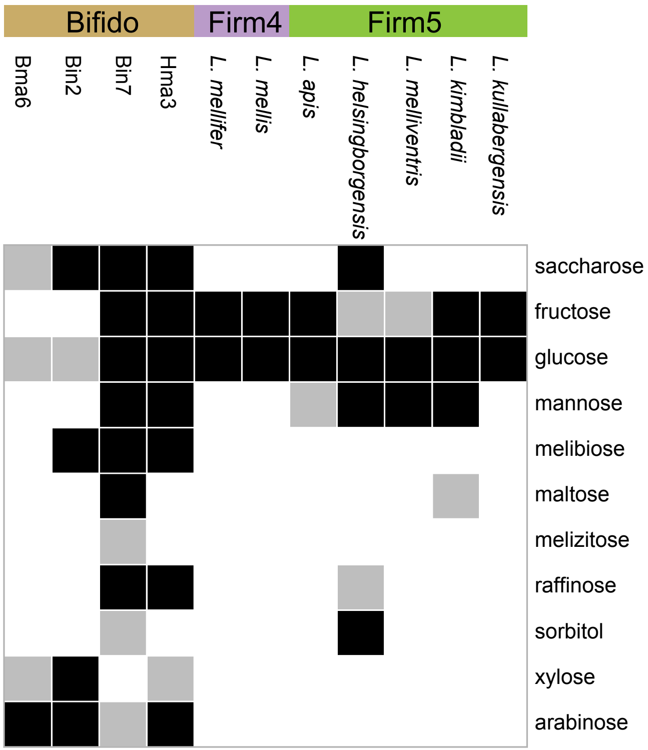
Bifido
Firm4
Firm5
Bma6
Bin2
Bin7
Hma3
L. mellifer
L. mellis
L. apis
L. helsingborgensis
L. melliventris
L. kimbladii
L. kullabergensis
saccharose
fructose
glucose
mannose
melibiose
maltose
melizitose
raffinose
sorbitol
xylose
arabinose
